# Supplementary material for: WNT-dependent interaction between inflammatory fibroblasts and FOLR2+ macrophages promotes fibrosis in chronic kidney disease
Source: Nat Commun. 2024 Jan 25;15:743. doi: 10.1038/s41467-024-44886-z (PMC10810789; doi:10.1038/s41467-024-44886-z)
Supplement: Supplementary file 1 — Supplementary Information [file 41467_2024_44886_MOESM1_ESM.pdf]

SUPPLEMENTARY INFORMATION for

WNT-dependent interaction between inflammatory fibroblasts and FOLR2+ macrophages promotes fibrosis in chronic kidney disease

Camille Cohen[1,2], Rana Mhaidly[1,2#], Hugo Croizer[1,2#], Yann Kieffer[1,2], Renaud Leclere[3], Anne Vincent-Salomon[3], Catherine Robley[1,2], Dany Anglicheau[4], Marion Rabant[5], Aurélie Sannier[6], Marc-Olivier Timsit[7], Sean Eddy[8], Matthias Kretzler[8,9], Wenjun Ju[8,9] and Fatima Mechta-Grigoriou[1,2,*]

# These authors contributed equally

[1] Institut Curie, Stress and Cancer Laboratory, Equipe labélisée par la Ligue Nationale contre le Cancer, PSL Research University, 26, rue d'Ulm, F-75248 Paris, France

[2] Inserm, U830, 26, rue d'Ulm, Paris, F-75005, France

[3] Department of Diagnostic and Theragnostic Medicine, Institut Curie Hospital Group, 26, rue d'Ulm, F-75248 Paris, France

[4] Department of Nephrology and Kidney Transplantation, Necker Hospital, AP-HP, Paris Cité University, Inserm U1151, 149 rue de Sèvres, 75015 *Paris, France*

[5] Department of Pathology, Necker Hospital, AP-HP, Paris Cité University, 149 rue de Sèvres, 75015 *Paris,* France

[6] Department of Pathology, AP-HP, Bichat-Claude Bernard Hospital, Paris Cité University, Inserm, U1148, 46, rue Henri Huchard, 75877, Paris, France

[7] Department of Urology, Européen George Pompidou Hospital, APHP, Paris Cité University, France.

[8] Department of Internal Medicine, University of Michigan, Ann Arbor, MI 48109, USA

[9] Department of Computational Medicine and Bioinformatics, University of Michigan, Ann Arbor, MI 48109, USA.

* Correspondence: Fatima Mechta-Grigoriou (ORCID Number: 0000-0002-3751-6989) Phone: +33 (0)1 56 24 66 53; E-mail address: fatima.mechta-grigoriou@curie.fr

Running title: Crosstalk of iFibro and FOLR2+ macrophages in CKD

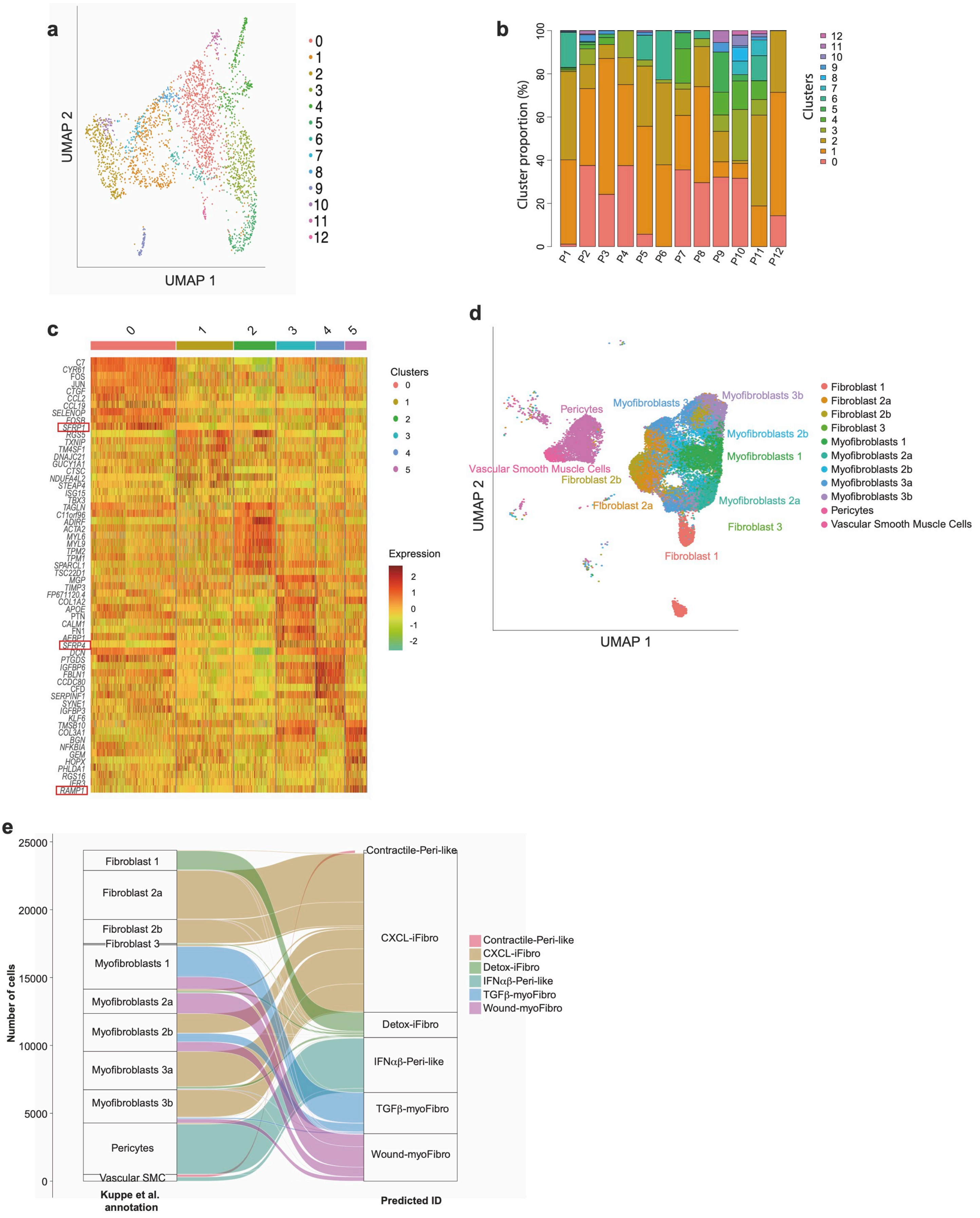

Supplementary Figure 1

Supplementary Figure 1: Characterization of differentially expressed genes in mesenchymal clusters

(**a**) UMAP of scRNAseq data from 2908 mesenchymal cells from {Kuppe, 2021 #4} across 12 patients suffering or not from CKD, allowing the visualization of 13 clusters (0 to 12). Colors show the different clusters defined by graph-based clustering method applied on the space defined by the 30 first principal components. (**b)** Barplot representing the proportion of cells of the 13 clusters (0 to 12) in each patient (P1 to p12). (**c**) Heatmap showing the expression of the top-10 most variable genes from the pairwise analysis performed between the different clusters of mesenchymal cells. (**d**) UMAP of scRNAseq data from mesenchymal cells from PDGFRβ+ sorted cells from {Kuppe, 2021 #4} using the original annotations from the paper. (**e**) Alluvial plot showing the result of the label transfer of data presented in (**d**) using our own annotations as reference. The left column represents the original annotations from {Kuppe, 2021 #4}, and the right column represents the annotations from our study.

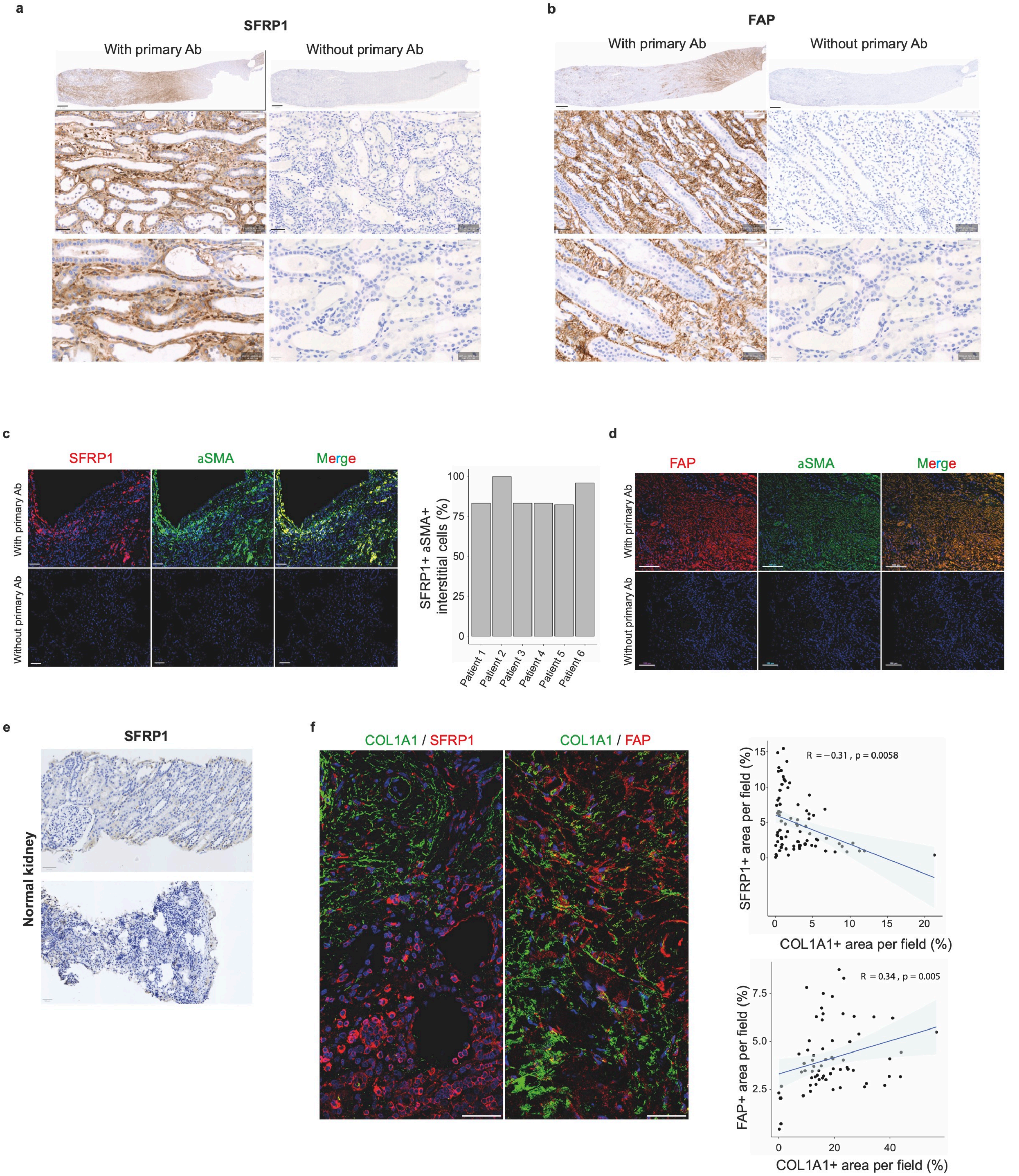

Supplementary Figure 2

Supplementary Figure 2: Characterization of SFRP1+ and FAP+ mesenchymal cells

(**a**) Additive representative images (in addition to those shown in **Fig. 2j, k**) to emphasize the stromal staining of SFRP1+ at low (upper panel) and high (lower panel) magnification. Left panel shows a representative image of the complete staining. The right panel shows the result of the staining using the same protocol without primary antibody, highlighting the specificity of the primary antibody. Scale bars = 1mm for the upper panel, 50µm for the middle panel, 20 µm for the lower panel. (**b**) Same as (**a**) but for FAP. Scale bars = 1mm for the upper panel, 50µm for the middle panel, 20 µm for the lower panel. (**c**) Representative images (left panel) and quantification (right panel) of IF showing co-staining between SFRP1 (red) and αSMA (green). Quantification shows the percentage of SFRP1+ αSMA+ double positive interstitial cells. N = 6 PKD patients. Low panels of images show the image resulting from the same protocol without primary antibody. (**d**) Same as in (**c**) but for FAP and αSMA co-staining. (**e**) Representative images of staining of SFRP1 in a normal kidney harvested from a kidney donor at the day of the transplantation. N=2 (males) different kidney donors. Scale bar =50µm. (**f**) Representative images (left panel) and quantification (right panel) of IF showing costaining between COL1A1 (green) and SFRP1 or FAP (red). Quantification represents the correlation between the percentage of COL1A1 positive area and SFRP1 (middle panel) or FAP (right panel) positive percentage area per surface unit, each dot representing a field of 6400μm^2 (80x80µm).

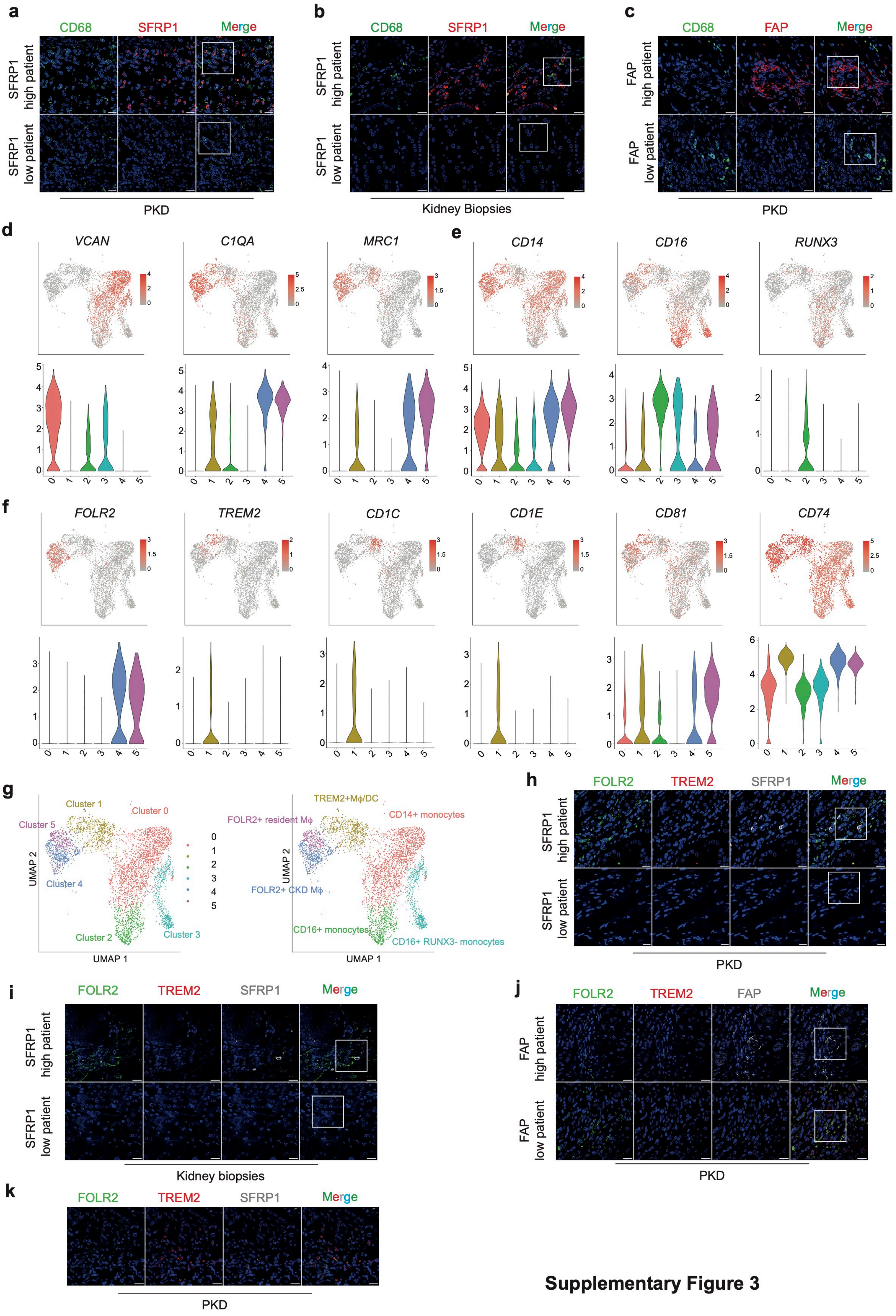

Supplementary Figure 3

Supplementary Figure 3: Fibroblasts / macrophages colocalization and myeloid cells characterization by single cell RNA sequencing

(**a**) Same representative images as in **Fig. 3a** of IF mono and co-staining of CD68 and SFRP1 (inflammatory fibroblast marker) in PKD patients (N = 6). SFRP1 high patient corresponds to a positive SFRP1 zone, in a patient with an elevated H-score for SFRP1, quantified in **Fig 2**. SFRP1 low patient corresponds to a negative SFRP1 zone, in a patient with a low H-score for SFRP1, quantified in **Fig. 2**. Scale bar = 20µm. N = 6 PKD patients. (**b**) Same as in (**a**) in fibrotic kidney biopsies (same images than in **Fig. 3b**). N = 10 patients with fibrotic kidney. (**c**) Same representative images as in **Fig. 3c** of IF mono and co-staining between CD68 and FAP (ECM-secreting myofibroblast marker) in PKD patients Scale bar = 20µm. N = 6 PKD patients. **(d)** UMAP (top) and violin plot (bottom) of scRNAseq data from 3960 myeloid cells from {Kuppe, 2021 #4} across 12 patients suffering or not from CKD, showing the expression of genes defining monocytes (*VCAN*) or macrophages (*C1QA* and *MRC1*). (**e**) Same as in (**d**) showing the expression of genes defining monocytes subtypes (*CD14*, *CD16* and *RUNX3*). (**f**) Same as in (**d**) showing the expression of genes defining macrophages (Mϕ) and dendritic cell (DC) subtypes (*FOLR2*, *TREM2, CD1C, CD1E, CD81* and *CD74*). (**g**) UMAP showing new annotations of the 6 myeloid cells clusters (0 to 5) identified in Control and CKD patients. (**h**) Same representative images than in **Fig. 3g** showing IF mono- and co-staining of FOLR2, TREM2 (markers of different macrophage subsets) and SFRP1 (inflammatory fibroblast marker) in PKD patients (N = 6). Scale bar = 20µm. (N = 6 PKD patients). (**i**) Same as in (**g**) in fibrotic kidney biopsies (Same representative images than in **Fig.3h**, N = 10 patients). (**j**) Same representative images than in **Fig. 3i** showing IF mono- and co-staining for FOLR2+ cells and FAP+ cells (ECM-secreting myofibroblasts) in PKD patients (N = 6). Scale bar = 20µm. (**k**) representative image of a co-staining between FOLR2, TREM2 and SFRP1, showing TREM2+ macrophages staining in PKD samples. Scale bar = 20 µm.

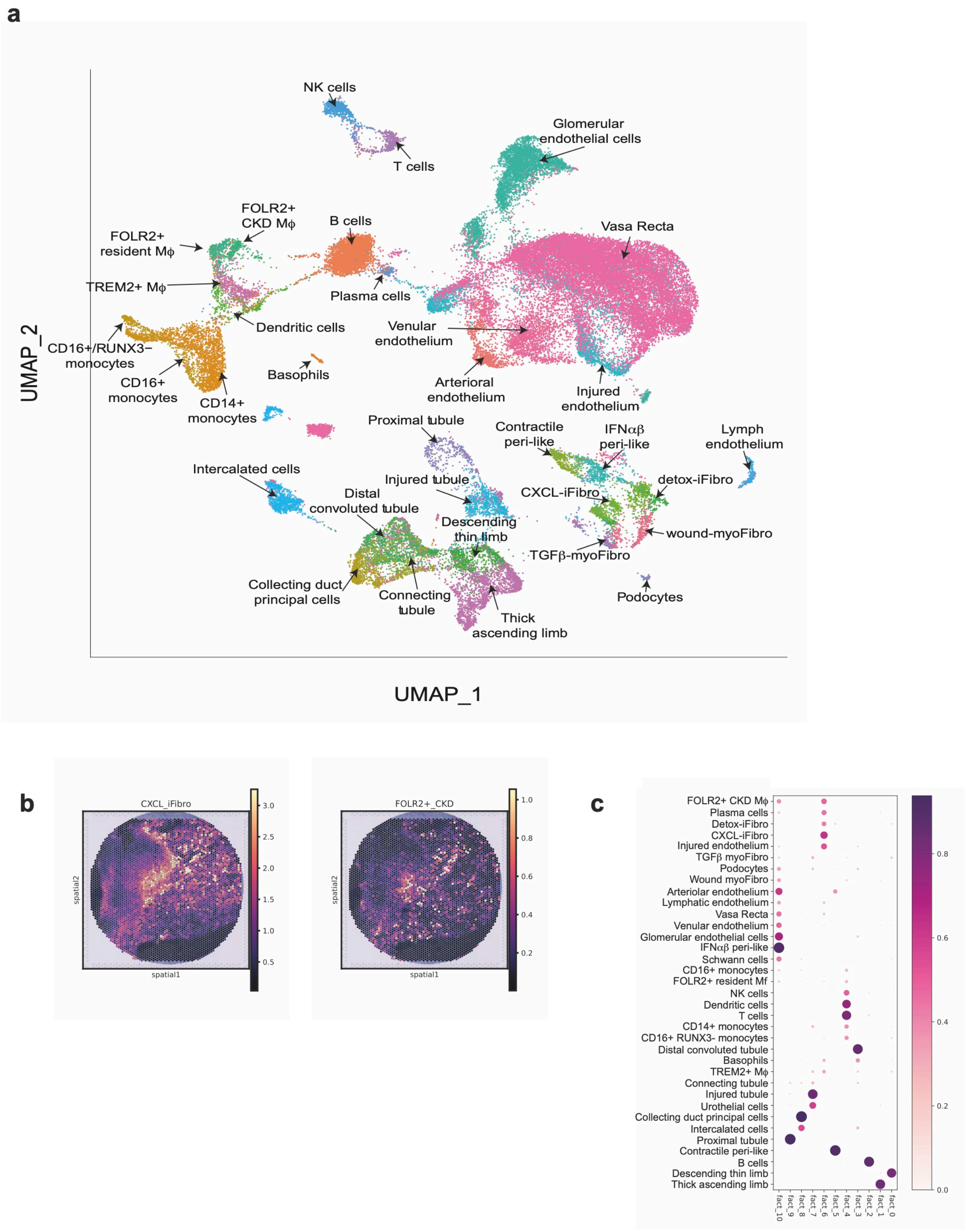

Supplementary Figure 4

Supplementary Figure 4: Single cell atlas and spatial transcriptomics analysis

(**a**) UMAP of scRNAseq data from 49 226 cells from {Kuppe, 2021 #4} across 12 patients suffering or not from CKD, identifying 35 cell types or state. (**b**) Results of the deconvolution for CXCL-iFibro and FOLR2-CKD macrophages using Cell2Location on patient #2 described in **Table S5**. For each cell type, the number of cells deconvoluted of the indicated cell type is plotted on the tissue. (**c**) Non-negative matrix factorization of the deconvolution output with 11 factors highlighting different microenvironment, and especially the colocalization between CXCL-iFibro and FOLR2-CKD macrophages. Color and size of the dots represent the proportion of cells of each cell type corresponding to each factor, normalized for the total abundance of the cell type.

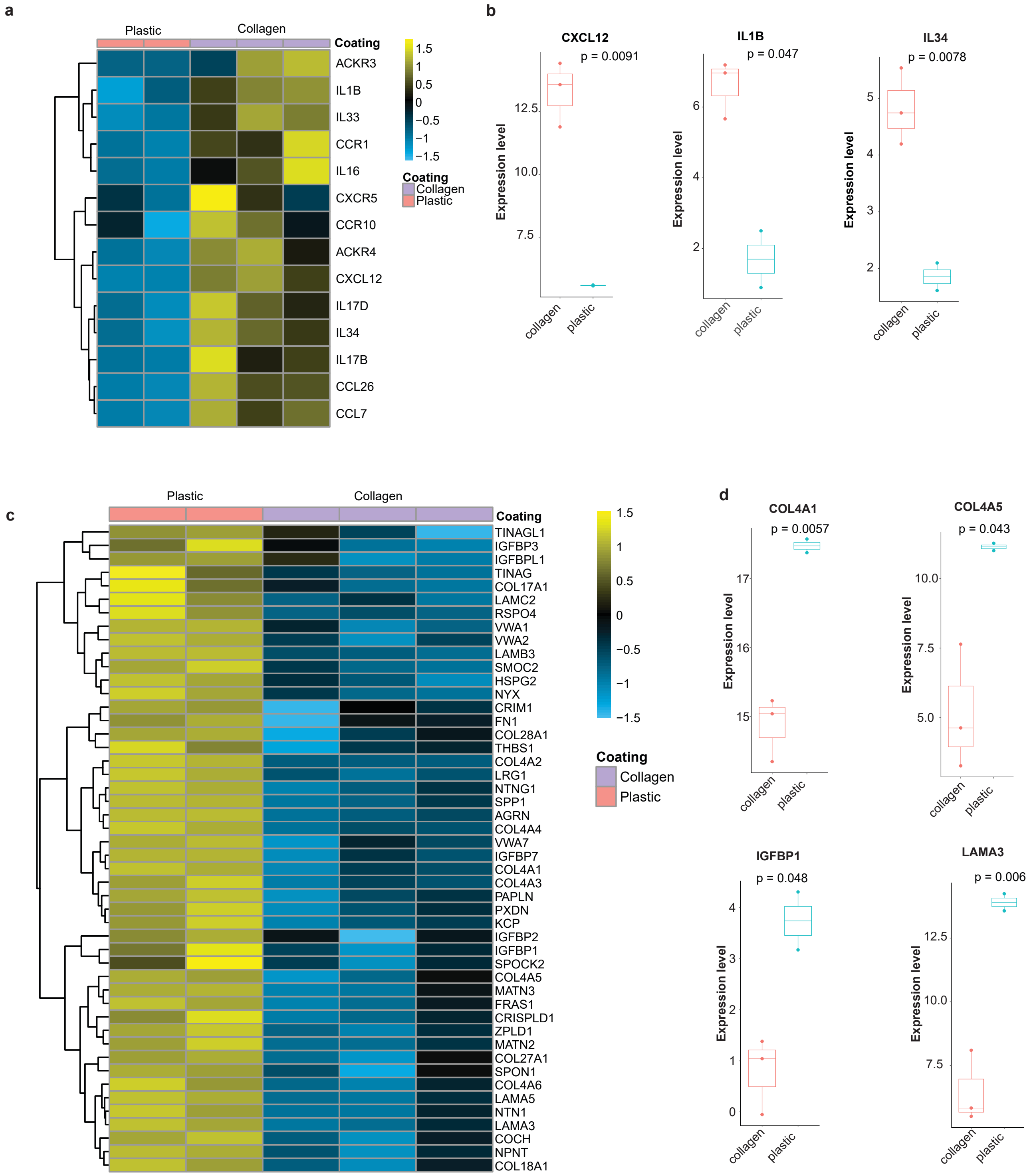

Supplementary Figure 5

Supplementary Figure 5: Characterization of primary fibroblasts cultured on collagen- or plastic-dishes.

(**a**) Heatmap showing the up-regulated inflammatory genes identified in collagen-cultured fibroblasts, with an adjusted p-value<0.05 (from DESeq2). Comparison between collagen-cultured (n = 3) *versus* plastic-cultured fibroblasts (n = 2). (**b**) Boxplots showing the normalized and log2-transformed expression from RNA-seq of *CXCL12*, *IL1B* or *IL34* in collagen-cultured (n = 3) *versus* plastic-cultured fibroblasts (n = 2). (**c**) Heatmap showing the up-regulated matrisome genes identified in plastic-cultured cells, with an adjusted p-value<0.05 (from DESeq2). Comparison between collagen-cultured (n = 3) versus plastic-cultured fibroblasts (n = 2). (**d**) Boxplots showing the normalized and log2-transformed expression from RNA-seq of *COL4A1*, *COL4A5*, *IGFBP1* and *LAMA3* in collagen-cultured (n = 3) *versus* plastic-cultured fibroblasts (n = 2).

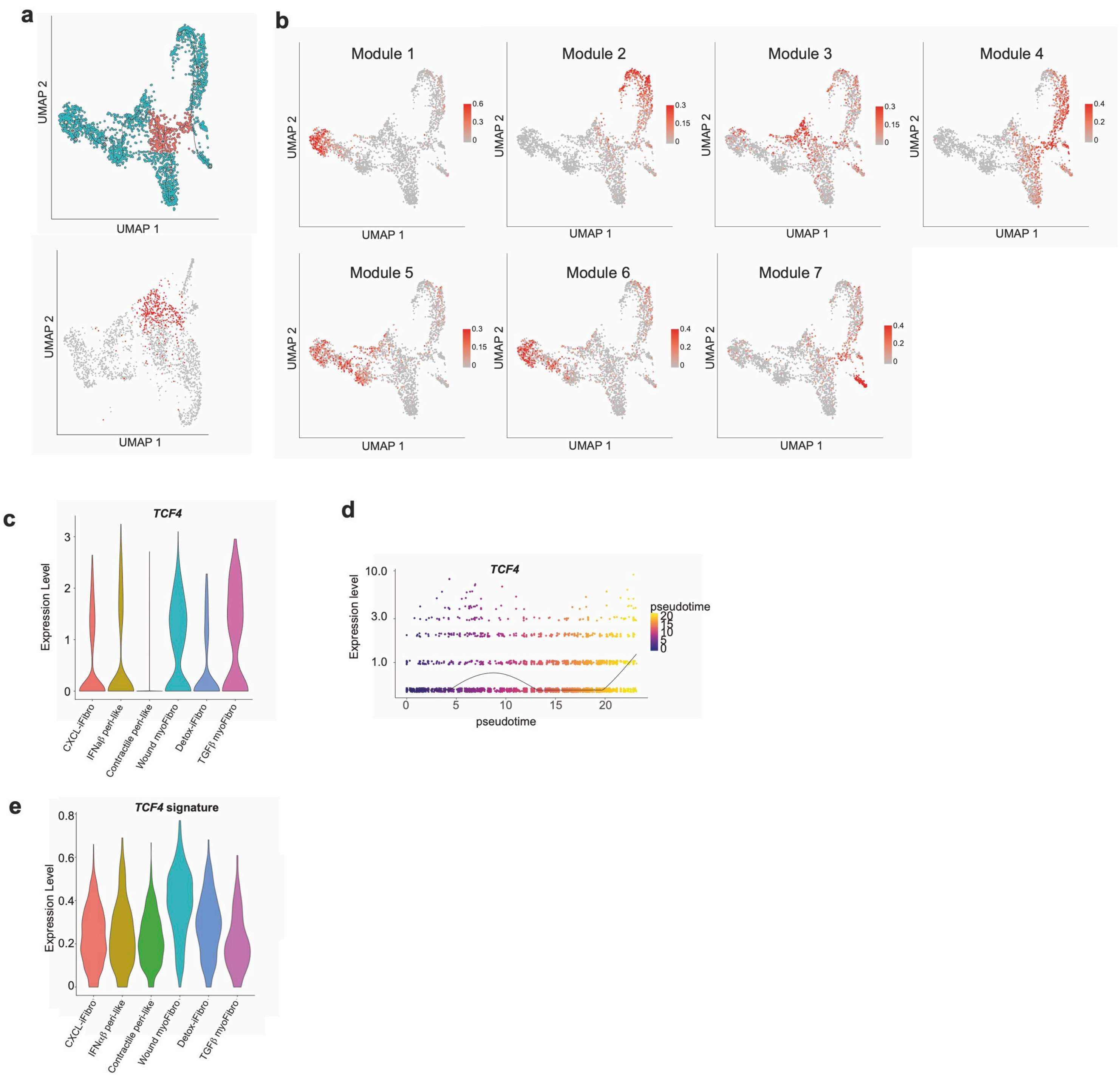

Supplementary Figure 6

Supplementary Figure 6: The Wnt-β-catenin pathway is involved in the switch from CXCL-iFibro to ECM-secreting myoFibro.

(**a**) Same UMAP as in **Fig. 2a** (upper UMAP) and in **Fig. 1a** (lower UMAP) indicating the subset of red cells for which the transcription factor inference analysis has been performed. (**b**) Same UMAP as in **Fig. 2a** showing the expression of the 7 different modules of coregulated genes identified by Monocle3. (**c**) Violin plot showing the expression of the transcription factor TCF4 in our different mesenchymal clusters. (**d**) Expression of the transcription factor TCF4 along pseudotime, as defined in **Fig. 2b.** (**e**) Violin plot showing the expression of TCF4-target genes (see list of genes **Table S13**) showed as a z-score.

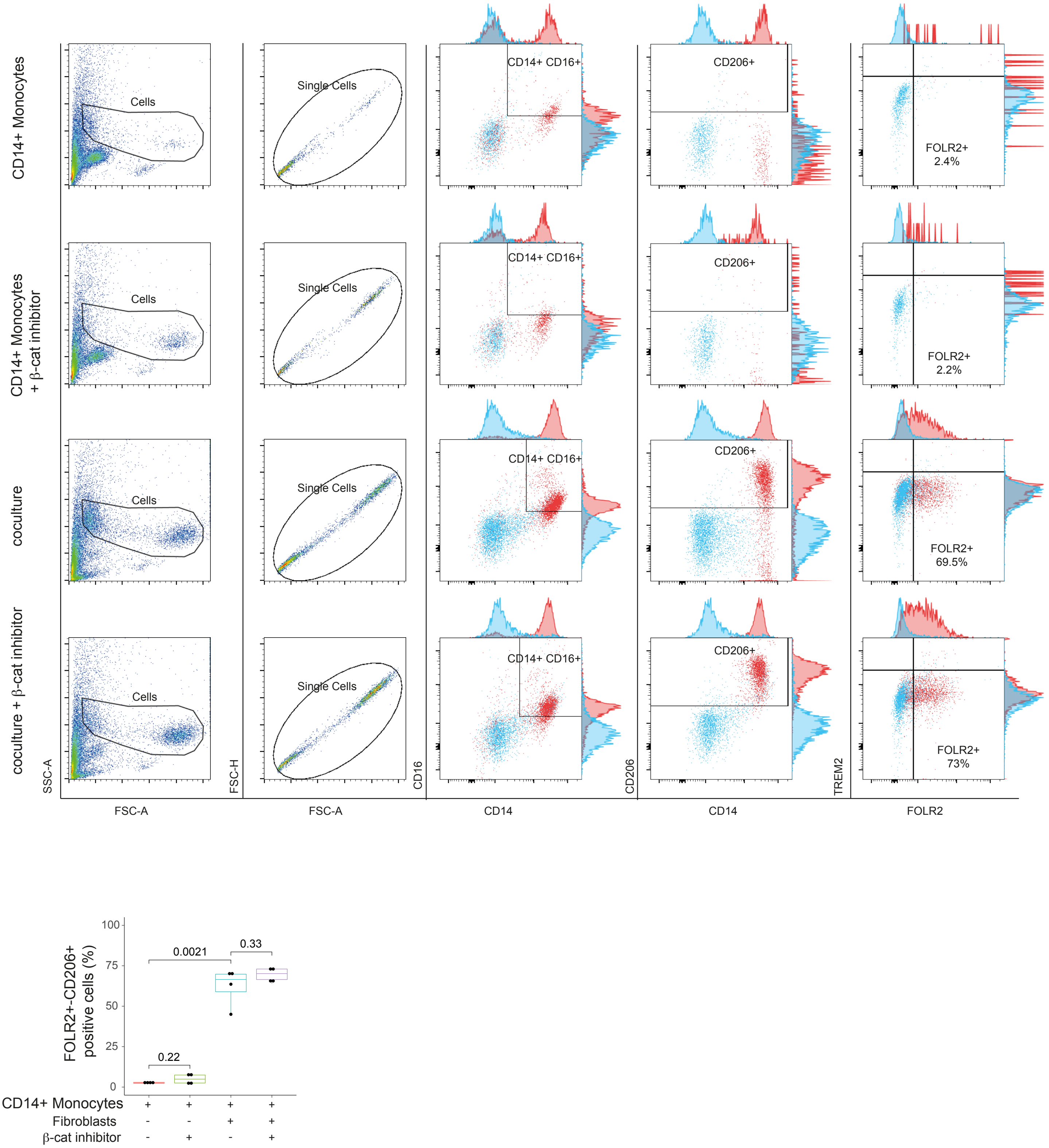

Supplementary Figure 7

Supplementary Figure 7: Macrophage polarization upon co-culture and treatment with β-catenin/TCF interaction inhibitor

Up, Representative plots and corresponding quantification of flow cytometry analysis aiming at characterizing macrophage phenotype after 24h of co-culture of CD14+ monocytes with collagen-cultured fibroblasts, with or without β-catenin/TCF interaction inhibitor. From left to right columns are represented FSC-A/SSC-A, FSC-A/FSC-H, CD14/CD16, CD14-CD206 and FOLR2/TREM2 expression. Bottom, Quantifications show the percentage of FOLR2+ CD206+ macrophages among alive CD14+ monocytes. P-values from Kruskall-Wallis test (n = 3 independent experiments with 3 different cell lines and 4 PBMC from healthy donors).

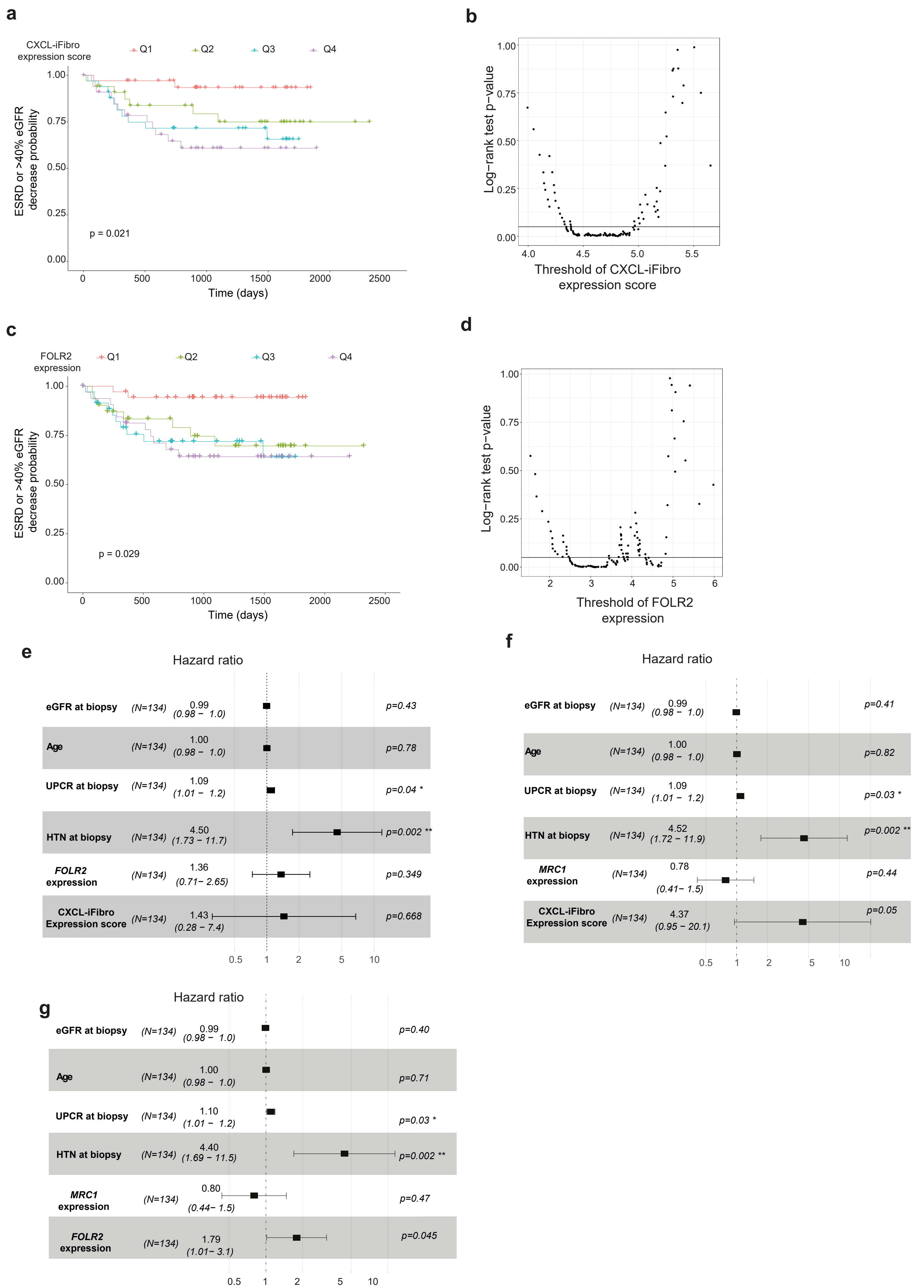

Supplementary Figure 8

Supplementary Figure 8: Multivariate analysis using Cox model integrating FOLR2 and CXCL-iFibro expression

(**a**) Kaplan-Meier curve showing the probability of the events composing the composite outcome (ESRD or loss of more than 40% of eGFR) according to the quartile of expression of CXCL-iFibro signature. N = 134 patients; with 31 events including 2 events in the lowest-expression quartile (Q1, N = 34), 7 in Q2 (N = 33), 10 in Q3 (N = 33) and 12 in Q4 (highest expression group, N = 34). (**b**) Scatter plot showing the p-value of the log rank test according to the threshold of CXCL-iFibro expression score selected for discrimination of patients (iterative method). Each dot represents a patient (n=134). (**c**) Same as (**a**), but for quartiles of FOLR2 expression. N = 134 patients; with 31 events including 1 event in the lowest-expression quartile (Q1, N = 34), 9 in Q2 (N = 33), 10 in Q3 (N = 33) and 11 in Q4 (highest expression group, N = 34). (**d**) Same as (**b**) but for FOLR2 expression in the x axis. (**e**) Forest plot showing the results of Cox multivariate analysis according to the following variables: eGFR at biopsy, age of the patient, urinary protein to creatinine ratio (UPCR), presence of hypertension, the expression score of CXCL-iFibro and FOLR2 expression. (**f**) same as (**e**) but with the following variables eGFR at biopsy, age of the patient, urinary protein to creatinine ratio (UPCR), presence of hypertension, the expression score of CXCL-iFibro score and the expression of MRC1. (**g**) same as (**f**) but with the following variables eGFR at biopsy, age of the patient, urinary protein to creatinine ratio (UPCR), presence of hypertension, FOLR2 expression and MRC1 expression.

Clusters	Category	Term	Description	LogP	Log(q-value)	InTerm_InList	Symbols
cluster 0 CXCL-iFibro	GO Biological Processes	GO:0043062	extracellular structure organization	-20.2369011145	-15.878	30/457	A2M,AEBP1,APOE,COL4A3,COL4A4,CST3,CCN2,CTSS,CYP1B1,DCN,FBLN1,ICAM1,CCN1,ITGA1,LOXL1,LUM,MFAP4,MMP19,NID1,PDGFRA,PLTP,SDC4,THBS1,VCAM1,ADAMTSL2,FBLN5,SPINT2,EMILIN1,EFEMP2,CRISPLD2,IGFBP3,IGFBP7,PRELP,SRGN,LTBP4,ABI3BP,SVEP1,EMID1
	GO Biological Processes	GO:0070848	response to growth factor	-16.3151848636	-12.558	32/736	CD63,CCN2,DCN,DUSP6,EGR1,FGF7,FOS,GPC3,HGF,NR4A1,CCN1,JUN,LUM,MYC,PDGFRA,PTN,CCL2,SFRP1,THBS1,CLEC3B,VCAM1,ZFP36,LTBP4,NRP1,ADAMTSL2,CITED2,FST,SPINT2,EMILIN1,SPART,ERRFI1,CCBE1
	GO Biological Processes	GO:0001944	vasculature development	-14.6410934433	-11.060	31/786	APOE,COL4A3,CCN2,CTSH,CYP1B1,DCN,TYMP,EGR1,GPC3,HGF,NR4A1,CCN1,JUN,LOXL1,MMP19,PDGFRA,PTGS2,PTN,CCL2,CCL11,SFRP1,SOD2,TCF21,THBS1,NRP1,SOCS3,ANGPTL1,EMILIN1,EFEMP2,ERRFI1,CCBE1,EDNRB,FGF7,IGFBP3,MYC,CXCL12,ZFP36,FST,SDC4,AKAP12,ARID5B
	Reactome Gene Sets	R-HSA-1280215	Cytokine Signaling in Immune system	-14.1322267897	-10.676	29/707	BIRC3,CEBPD,CNN2,DUSP6,EGR1,FOS,GBP1,HGF,ICAM1,IL6ST,JUN,LIFR,MYC,PIM1,PTGS2,CCL2,CCL11,CCL19,SOD2,VCAM1,IFITM1,SOCS3,OSMR,IL32,IFITM3,IFITM2,SAMHD1,IL33,IL34,CNTN1,COL4A3,COL4A4,NR4A1,ZFP36,SDC4,NFKBIZ,DUSP1,HMOX2,CCN2,FHL2,PDGFRA,NRP1,C7,CXCL12,CCN1
Cluster 1 IFN-Pericyte-like	Reactome Gene Sets	R-HSA-909733	Interferon alpha/beta signaling	-9.9545467334	-5.596	10/70	IFI6,HLA-A,HLA-B,HLA-C,HLA-F,IFI27,IFIT3,IFITM1,ISG15,IFITM3,CD44,MT2A,CD36,MEF2C,PSME1,TNFSF12,KCNJ8,ABCC9,IFI44L,GAPDH,ADAMTS5,STOM
	GO Biological Processes	GO:0001568	blood vessel development	-8.6123307162	-4.817	24/754	JAG1,AGTR1,RHOB,BTG1,EPAS1,FOXS1,GJA4,HES1,ID1,MEF2C,NGFR,PDGFRB,PGF,PTK2,SPARC,TBX2,NR2F2,TGFBI,TNFSF12,RAPGEF2,GJC1,ADGRA2,APOLD1,ADGRF5,PTP4A3,ZEB2,WFDC1,ARHGDIB
	GO Biological Processes	GO:0022604	regulation of cell morphogenesis	-8.3209780067	-4.806	19/486	RHOB,RHOC,CD44,SEPTIN7,DPYSL2,EPS8,ID1,ITGA7,MAP1B,NGF,NGFR,PTK2,SPARC,EZR,RAPGEF2,DLC1,ARHGAP15,FGD4,WTIP,ARHGDIB,KCNJ8,MYO1B,PAWR,PDGFRB,NCK2,CDC42BPA,ARHGEF17,GJC1,PACSIN2,SYNE2,KCNE4,EHD2,ESAM,FRMD3,NES,CD36,TPPP3
Cluster 2 Pericyte-like	KEGG Pathway	ko00190	Oxidative phosphorylation	-27.3073122464	-23.339	32/133	ATP5F1E,ATP5MC2,ATP5ME,ATP5PF,ATP6V1E1,ATP5PO,COX5B,COX6A1,COX6C,COX7A1,COX7A2,COX7B,COX7C,COX8A,NDUFA4,NDUFA5,NDUFA7,NDUFB1,NDUFB7,NDUFB10,NDUFC1,NDUFS5,NDUFS6,NDUFS8,NDUFV2,UQCRB,COX17,UQCR11,UQCRQ,UQCR10,NDUFA4L2,COX4I2
	GO Biological Processes	GO:0061061	muscle structure development	-27.2207700382	-23.339	60/632	JAG1,ZFHX3,CAV1,CAV2,CD9,CFL2,CRYAB,CSRP1,CSRP2,DMD,DMPK,ELN,EPAS1,FOXC2,HES1,ID2,ID3,IGFBP5,ITGA7,LAMA5,BORCS8-MEF2B,MEF2C,MEF2D,MYH11,MYL6,MYLK,PGM5,SORT1,SGCA,TAGLN,TBX2,TPM1,UTRN,BTG2,KCNAB1,FZD7,SORBS2,YBX3,MYOM1,CACNA1H,USP2,PDLIM1,PDLIM7,AKAP6,HDAC9,WDR1,HDAC5,SPEG,LDB3,HEY2,LMOD1,NUPR1,HEYL,EHD2,COPRS,SOX6,JPH2,MYOZ1,MYOCD,RBPMS2,NDUFV2,PLN,CDKN1A,CRIP1,EDNRA,FLNA,ITGA3,NTRK3,PTK2,ROCK1,HEXIM1,HSPB7,PLCE1
	GO Biological Processes	GO:0003012	muscle system process	-26.8771095813	-23.120	51/446	ACTA2,ALDOA,ATP1A2,ATP2B4,CALM2,CALM3,CAMK2G,CASQ2,CAV1,CKMT2,CNN1,CRYAB,DAPK3,DES,DMD,DMPK,EDNRA,FLNA,IGFBP5,ITGA2,KCNA5,KCNMA1,MYH11,MYL6,MYLK,PPP1R12B,PGAM2,PIK3C2A,PLN,PRKG1,ROCK1,SGCA,SNTA1,TBX2,TPM1,TPM2,UTRN,SORBS2,MYOM1,CACNA1H,NOL3,AKAP6,SORBS3,MYL9,SYNM,HEY2,LMOD1,PLCE1,TMEM38B,MYOZ1,MYOCD,APP,ATP1B2,FXYD2,EPAS1,GNAS,HRH2,JAK2,NPR1,MGLL,HSPB7,C1QTNF1,FBXO32,ADCY6,SLC25A4,GUCY1B1,BORCS8-MEF2B,MEF2C,MEF2D,PPP1R12A,PDE3A,PPP1CB,PDE5A,IRAG1,ELN,FOXC2,HOXB2,ID2,KLF2,AOPEP,ACTB,ACTN4,OAZ2,PTPN3,RRAD,SCN3A,CX3CL1,YWHAH,KCNAB1,WDR1,COX17,YWHAQ,JPH2,TSPO,DBI,FABP3,SNCG,INPP5A,SCTR,RCAN2,CAP2,CAP1,NUDT4,CAMTA1,ADGRL3,ACKR3,PDE4C,ATP6V1E1,CAV2,GPR20,MT1E,MT1M,MT1X,MT2A,PDK4,SKP1,PICALM,NCS1,RAB20,NTRK2,ATP10A,CDS2,SUN1,SUN2,GJA4,ANO3,CABP1,PKIG,PACSIN2,PNKD,TMEM14A,FRMD4A,MIDN
	GO Biological Processes	GO:0030029	actin filament-based process	-24.4433827959	-20.930	63/789	ACTB,ACTN4,ACTN1,ALDOA,RHOB,ATP1A2,CAPZA2,CAV1,CFL2,CNN1,CSRP1,CSRP2,DAPK3,DES,DMD,ELN,FLNA,HIP1,ID1,JAK2,KCNA5,MYH11,MYL6,MYO1C,NTRK3,PDGFA,PFN1,PGM5,PLN,PLS3,PRKG1,ROCK1,CX3CL1,SNTA1,TPM1,TPM2,SORBS2,BRSK2,PDLIM1,PDLIM7,ARHGEF17,WDR1,ARPC5,ARPC2,SORBS3,CAP1,ARPC1A,DSTN,LDB3,PACSIN2,SYNPO,LIMCH1,SYNE2,SUN2,LMOD1,RGCC,EHD2,EPDR1,ARHGEF10L,MYOZ1,INF2,ESAM,EHBP1L1,APP,CLU,CRYAB,FKBP4,FOXC2,TPPP3,PTK2,DYNLT1,NES,PLK2,MAPRE2,FBXW5,NTRK2,PLCE1,AMIGO2,YWHAH,YWHAQ,TOMM7
	KEGG Pathway	ko04260	Cardiac muscle contraction	-15.2495582205	-12.581	18/78	ATP1A2,ATP1B2,FXYD2,COX5B,COX6A1,COX6C,COX7A1,COX7A2,COX7B,COX7C,COX8A,TPM1,TPM2,UQCRB,UQCR11,UQCRQ,UQCR10,COX4I2,ACTN4,ATP2B4,ATP5F1E,ATP5MC2,ATP5ME,ATP5PF,ATP6V1E1,ATP5PO,CASQ2,CAV1,DMD,DMPK,FLNA,KCNA5,KCNMA1,NDUFA4,PTPN3,SCN3A,SNTA1,UTRN,YWHAH,KCNAB1,CACNA1H,AKAP6,COX17,TMEM38B,NDUFA4L2,SLC38A1,SLC38A11,CALM2,CALM3,PLN,RRAD,CX3CL1,NOL3,NCS1,JPH2,PNKD,HIGD1A,COX14,LAMTOR3,YWHAQ,DDIT4,NDUFS8,CDKN1A,BTG2,USP2,PLK2,RGCC
Cluster 3 Wound-myoFibro	Canonical Pathways	M5884	NABA CORE MATRISOME	-62.5579191225	-58.199	66/275	AEBP1,COL1A1,COL1A2,COL3A1,COL4A1,COL4A2,COL4A4,COL5A1,COL5A2,COL6A1,COL6A2,COL6A3,COL8A1,COL12A1,COL15A1,COL16A1,COMP,VCAN,DCN,ECM1,FBLN1,FBLN2,FBN1,EFEMP1,FN1,HSPG2,IGFBP4,IGFBP6,IGFBP7,LAMA2,LAMB1,LAMB2,LTBP1,LTBP2,LTBP3,LUM,MATN2,MFAP4,MGP,CCN3,OMD,OGN,PCOLCE,PRELP,SPARC,THBS2,TNXB,COL14A1,SPARCL1,SRPX,LTBP4,SLIT2,EDIL3,LAMC3,SPON2,SPON1,FBLN5,POSTN,EMILIN1,MXRA5,ABI3BP,ASPN,COL21A1,HMCN1,PAPLN,CTHRC1,A2M,APOE,CD34,CST3,CTSK,CYP1B1,ENG,FAP,GSN,ITGAV,ITGB1,ITGB5,LRP1,MMP2,MMP14,DDR2,P4HB,SERPINE1,PDGFRA,PLTP,HTRA1,TGFB1,TIMP1,TIMP2,MMP23B,ADAMTSL2,PHLDB1,SULF1,FLRT2,ANTXR1,CCDC80,DST,PPIB,SDC2
	Reactome Gene Sets	R-HSA-3000178	ECM proteoglycans	-32.4025808511	-28.822	28/76	COL1A1,COL1A2,COL3A1,COL4A1,COL4A2,COL4A4,COL5A1,COL5A2,COL6A1,COL6A2,COL6A3,COMP,VCAN,DCN,FN1,HSPG2,ITGAV,ITGB1,ITGB5,LAMA2,LAMB1,LAMB2,LUM,SERPINE1,SPARC,TGFB1,TNXB,ASPN,A2M,COL8A1,COL12A1,COL15A1,COL16A1,CTSK,FBN1,MMP2,MMP14,HTRA1,TIMP1,TIMP2,COL14A1,DDR2,SDC2,LAMC3,MDK,TGM2,THBS2,P4HB,PCOLCE,PPIB,COL21A1,DST,PIK3R1,EDIL3,CCND2,PAK3,PDGFRA,PDGFC,PDGFD,MYL12B,HMCN1,PAPLN,F2R,FGFR1,HSP90B1,PELO,CREB3L2,AEBP1,CYP1B1,EMILIN1,NOTCH2,PKM,EIF2AK2,WNT2B,FZD7,ANTXR1,PRNP,SPTAN1,SPTBN1,APOE,CALR,LRP1,SSC5D,CALM1,MEF2A,FLRT2,IL1R1,CNTN1,CFH,MITF,SNAI2,FMNL2,SCARB2,DAB2,CD55,GOLGA2,MYH9,ITSN1,RAB31,SEC31A,GOLIM4,SNX9,ACBD3,SH3D19
	GO Biological Processes	GO:0001568	blood vessel development	-25.5953060216	-22.081	57/754	AHR,ANXA1,ANXA2,APOE,CD34,CDH13,COL1A1,COL1A2,COL4A1,COL4A2,COL5A1,COL8A1,COL15A1,COMP,CYP1B1,DCN,ECM1,ENG,FAP,FN1,GPC3,HSPG2,ITGAV,ITGB1,LRP1,MDK,MMP2,MMP14,MYH9,NOTCH2,CCN3,SERPINE1,PDGFRA,SERPINF1,PKM,PRRX1,PTGIS,PTN,RORA,SPARC,TCF21,TGFB1,THBS2,THY1,SLIT2,RAMP1,GPNMB,SEMA3C,EMILIN1,SULF1,JCAD,PDGFD,ANTXR1,MTDH,PRICKLE1,CLEC14A,UNC5B
	GO Biological Processes	GO:0097435	supramolecular fiber organization	-22.602944354	-19.285	52/713	ADD3,AEBP1,APOE,DST,COL1A1,COL1A2,COL3A1,COL5A1,COL5A2,COL12A1,COMP,CSRP1,CST3,CYP1B1,DPYSL3,FKBP1A,GOLGA2,GSN,ITGB1,ITGB5,LTBP2,LTBP3,LUM,MARCKS,MAP1A,MAP1B,MEF2A,MFAP4,MYO1D,DDR2,PAK3,PDGFRA,PIK3R1,SPTAN1,SPTBN1,SVIL,TAC1,TNXB,HSP90B1,COL14A1,VIM,TMSB10,SLIT2,MICAL2,ARPC1B,FBLN5,CDC42EP3,EMILIN1,RUFY3,SNX9,ENAH,ARHGAP18,ANXA1,BST2,CALR,EPB41L2,EPHA3,LRP1,MDK,MYH9,MYH10,NOTCH2,PAM,MRAS,DAAM1,PALLD,ANTXR1,FMNL2,ATP2B1,CRABP2,F2R,FN1,HTR2B,SEMA3C,MACF1,DEPTOR,PHLDB1,NEXN,GPX4,LGALS3,MITF,EIF2AK2,TGFB1,LMO4,DACT1
	GO Biological Processes	GO:0009611	response to wounding	-20.5009818987	-17.318	49/701	A2M,ANXA1,ANXA5,APOE,DST,CD34,COL1A1,COL1A2,COL3A1,COL5A1,COMP,CSRP1,DCN,DPYSL3,F2R,FAP,FBLN1,FN1,GAP43,GSN,INHBA,ITGB1,LAMB2,LRP1,MAP1B,MDK,MYH9,NOTCH2,CCN3,SERPINE1,PDGFRA,SERPINE2,PIK3R1,PKM,PPP3CA,PROS1,PTN,SPARC,TFPI,TGFB1,TIMP1,FZD7,CPQ,POSTN,MACF1,FGFR1OP2,MYOF,CREB3L2,DCBLD2,PAM,TAC1,CD99
Cluster 4 Detox-iFibro	Canonical Pathways	M5884	NABA CORE MATRISOME	-29.2202873356	-24.861	36/275	AEBP1,COL6A1,COL6A2,DCN,DPT,FBLN1,FBLN2,FBN1,EFEMP1,GAS6,IGFBP3,IGFBP6,CCN1,LAMA4,MATN2,MFAP4,MGP,OMD,OGN,PCOLCE,PRELP,SPOCK1,TNXB,COL14A1,MFAP5,SRPX,LTBP4,SLIT2,FGL2,ABI3BP,PCOLCE2,NDNF,THSD4,SVEP1,RSPO3,PODN,CST3,CTSK,CYP1B1,GSN,LRP1,MMP2,PLTP,HTRA1,SDCBP,SFRP2,TIMP2,ADAMTS3,ANGPTL7,RAMP2,FLRT2,CCDC80,SDC2
	KEGG Pathway	hsa04610	Complement and coagulation cascades	-16.6847522146	-13.104	17/91	CFB,SERPING1,C1R,C1S,C3,C4A,C4B,C7,CLU,AKR1C1,CFD,F3,F10,CFH,CFI,PROS1,PROCR,CD81,AEBP1,ASPH,CES1,CPE,CTSH,GAS1,GSN,IGKC,PRNP,CLEC3B,ADAMTS3,CPXM2,FBLN1,IGHA1,MFAP4,DCN,HLA-DPA1,HLA-DRB1,SELENOP,PLSCR1,NUPR1,CXCL2,RARRES2,SLPI,CTSK,GBP1,LGALS3,PAK3,PIK3R1,UBC,NR4A3,RTN4,ANXA1,APOD,IGF1,MDK,SERPINF1,KLF4,PLD3,H19,CD74,HTRA1,XBP1,FGL2,TNFSF13B,CXCL12,VIM
	GO Biological Processes	GO:0030155	regulation of cell adhesion	-16.456928793	-12.943	38/734	ANXA1,APOD,CD74,CD81,CYP1B1,DUSP1,FBLN1,FBLN2,GBP1,GSN,HLA-DPA1,HLA-DRB1,IGF1,IGF2,CCN1,LAMA4,LGALS3,LRP1,MDK,AFDN,PAK3,PIK3R1,PRNP,S100A10,CXCL12,SFRP1,SFRP2,SPOCK1,XBP1,NR4A3,PLPP3,KLF4,GPNMB,TNFSF13B,FGL2,ADAM22,NDNF,CCDC80,FGF10,GAS6,IGHA1,IGKC,PLSCR1,SERPING1,FBN1,MYC,HTRA1,SLIT2,TRIB1,SAMHD1,C3,CLU,DDIT3,F3,PLD3,ERRFI1,H19,ITM2A,KLF10,CES1
	GO Biological Processes	GO:1901652	response to peptide	-10.8922475962	-8.177	26/526	ANXA1,KLF5,FBN1,GJA1,NR4A1,IGF1,IGF2,LRP1,MMP2,NR4A2,SERPINF1,PIK3R1,PRNP,RARRES2,CXCL12,TGFBR3,KLF10,VIM,XBP1,BTG2,NR4A3,TNFSF10,KLF4,NAMPT,ERRFI1,SESN3,COL6A1,SFRP1,SLC8A1,SLIT2,PDGFD,CTSH,AKR1C1,DUSP1,PLPP1,RAMP2
Cluster 5 TGFβ-myoFibro	Reactome Gene Sets	R-HSA-156842	Eukaryotic Translation Elongation	-41.4630624674	-37.104	36/93	EEF1G,RPL3,RPL4,RPL7,RPL12,RPL17,RPL18A,RPL21,RPL27A,RPL31,RPL32,RPL34,RPL35A,RPL37A,RPL38,RPL39,RPL41,RPL36A,RPLP0,RPLP1,RPLP2,RPS2,RPS6,RPS14,RPS15A,RPS17,RPS18,RPS19,RPS20,RPS27,RPS28,RPS29,RPL35,RPL13A,RPL36,RPS27L,EIF3J,CEBPB,TRAM1,SEC61G,HERPUD1,KDELR2,KDELR3,SLC25A6,HSPA1B,POLR2L,TGFB1,NR4A2,RSL24D1,MRPS6,PSMA2,PSMA4,ELOB,ABL2,PFN1,CXCR4,SMARCB1,LEF1,ACTB,CACNA1D,COL3A1,COL4A1,COL4A2,COL5A1,COL5A2,COL6A3,DPYSL3,EGR2,MMP2,NAB2,SDC2,PDLIM7,ARPC1B,UNC5B,HSPB1,TMED2,NOP10,ISG20L2,NAP1L1,BTG2,DIO2,SAT1,TDO2,DUOX1,CHDH,AZIN2,ATOX1,DNAJB1,CXCL8,MT1F,MT2A,RHEB,TXN,UBE2D3,BAG2,HSPH1,LAMTOR2,BAX,PMAIP1,RAB31,ROMO1,PNPT1,NFKBIA,LAMP5,RAB23,UBL5,SLIRP,ARF6,GLRB,MMP14,THY1,NRXN2,RAMP1,NKD2,ENTPD1,PDE4B,ENPP1,SPP1,ENTPD4,MOXD1,IGFBP5,SOX4,ENC1,TMA7,SNRPD2
	GO Biological Processes	GO:0030198	extracellular matrix organization	-29.3447955512	-26.566	48/395	BGN,RUNX1,SERPINH1,COL1A1,COL1A2,COL3A1,COL4A1,COL4A2,COL5A1,COL5A2,COL6A3,COL7A1,COL8A1,COL10A1,COL15A1,COL16A1,COMP,VCAN,CCN2,CTSK,CYP1B1,ENG,FAP,FN1,B4GALT1,HAS2,TNC,LOXL1,LUM,MFAP2,MMP2,MMP11,MMP14,SERPINE1,SFRP2,FSCN1,SPARC,SPP1,TGFB1,TIE1,TIMP1,ADAM12,ADAMTSL2,LAMC3,POSTN,SULF1,FKBP10,ANTXR1,CTSB,PCOLCE,PPIB,SDC2,ASPN,PLAU,TGM2,THBS2,ACTB,ARF6,BAX,CTNNB1,EGR2,HSPB1,JUNB,STMN1,NAB2,PLAT,POLR2L,SGK1,SPHK1,ELMO1,TRIB1,PDGFC,FXYD2,FTH1,JCHAIN,HSPH1,THY1,CXCL8,CCL2,XIAP,PPP1CC,F2R,RHEB,RPS6,LPAR6,CACNA1D,HES1,WNT5A,NFKBIA,CD74,MIF
	Canonical Pathways	M5884	NABA CORE MATRISOME	-28.625748381	-25.880	41/275	BGN,COL1A1,COL1A2,COL3A1,COL4A1,COL4A2,COL5A1,COL5A2,COL6A3,COL7A1,COL8A1,COL10A1,COL15A1,COL16A1,COMP,VCAN,CCN2,ECM1,FN1,TNC,IGFBP2,IGFBP5,LUM,MFAP2,MGP,PCOLCE,SPARC,SPOCK1,SPP1,THBS2,TNFAIP6,LAMC3,SPON2,SPON1,POSTN,SRPX2,ASPN,FNDC1,CTHRC1,VWCE,HMCN2
	GO Biological Processes	GO:0009611	response to wounding	-14.1742992066	-11.718	42/701	ACTB,ANXA5,ARF4,BAX,RUNX1,ENTPD1,COL1A1,COL1A2,COL3A1,COL5A1,COMP,CCN2,DPYSL3,F2R,FAP,FN1,B4GALT1,HSPB1,TNC,INHBA,LGALS1,SERPINE1,SERPINE2,PLAT,PLAU,PTK7,SPARC,SPP1,TGFB1,TIMP1,WNT5A,CXCR4,TFPI2,KLF4,NREP,POSTN,ABHD2,TNFRSF12A,DUOX1,DOCK6,FKBP10,TRIM72,HAS2,STMN1,PAM

Supplementary Table S1: Functional enrichment results of the 6 clusters of mesenchymal cells identified in **Fig. 1** characterized by the Metascape tool. The p-value is the result of a hypergeometric enrichment test. Q-value correspond to the Bonferonni corrected p-value (or False Discovery Rate).

CAF_S4_signature	
CASQ2	C20orf202
ADAP2	TRPC4
HIGD1B	SLC38A11
NRARP	SCN3A
KCNA5	RGS6
ESAM	RERGL
CDH6	PDE5A
TMEM74B	NEURL1B
AVPR1A	PLCE1
COX4I2	SYNPO2
PPP1R14A	KCNMB1
ARHGAP15	FMO3
OR51E2	PCDH1
OR51E1	AGAP11
GJA4	SSTR2
AKAP6	GPR116
FHL5	EPS8L1
GPR20	ARHGAP44
WFDC1	DGKG
CACNA1H	HEYL
ATP1A2	ACTC1
DPY19L2	NPY1R
SLC2A4	TRPC6
RBM20	ACTG2
CD93	CAPN12
PI15	RBPMS2
TPSG1	SCN4B
P2RY14	RYR2
PDGFB	LMOD1
SLCO1C1	OSTBETA
SH2D3C	CDH8
KCNK17	ENPEP
KIAA1274	JPH2
RASGRP2	GUCY1A2
LINGO1	GUCY1B3
NRIP2	FAM13C
ANGPT2	CSRNP3
C4orf32	CRHR2
LRRC10B	ACAN
SEMA5B	RASL12
SEPT4	GPR4
AOC3	HRC
CCDC141	CCDC102B
SGCA	KCNJ8
CD4	PHYHIPL
C21orf7	RASD2
SPTB	VSIG2
MYOM1	SLC8A1
DGKB	
GPR39	
KLHL30	
NFASC	

Supplementary Table S2: list of genes composing the CAF-S4 signature.

Percentages (%)	Contractile-Peri_like	CXCL-iFibro	Detox-iFibro	IFNαβ-Peri-like	TGFβ-myoFibro	Wound-myoFibro
Fibroblast 1	0.5	0.3	75.7	0	0.1	1
Fibroblast 2a	0	30.5	0.4	0.2	0	0.8
Fibroblast 2b	0	14.9	0.1	0.2	0.2	0
Fibroblast 3	0	0	5.1	0	0	0
Myofibroblasts 1	0.5	0.9	0	0.5	73.9	25.6
Myofibroblasts 2a	0	1.1	7.5	0	0.5	43.1
Myofibroblasts 2b	0	12.3	0.6	0.2	20.6	20.2
Myofibroblasts 3a	0	22.1	9	0	1.4	0.6
Myofibroblasts 3b	0	17.2	1.5	0.3	3.1	8.6
Pericytes	4.8	0.6	0.2	91.1	0.2	0.1
Vascular Smooth Muscle Cells	94.2	0	0	7.5	0	0

Supplementary Table S3: Table showing the percentages of correspondence between original mesenchymal cell type annotations from[8] and our annotations.

Clusters	Category	Term	Description	LogP	Log(q-value)	nTerm_InLis	Symbols
cluster 5 FOLR2 resident	Reactome Gene Sets	R-HSA-9012999	RHO GTPase cycle	-15.35441664	-11.006	41/449	ARHGAP4,ARHGAP5,DST,CLTC,STOM,HSP90AA1,HSP90AB1,KTN1,LMAN1,NCF4,PIK3R1,PKN2,USP9X,NCK2,ROCK2,SLC4A7,CDC42BPB,DOCK4,SRGAP3,FAM13A,NISCH,ANKRD26,PLXND1,CYFIP1,SRGAP2,PHIP,UACA,PLEKHG5,SRGAP1,FAM135A,PREX1,ARHGAP21,DOCK7,ARHGAP18,XPO1,ABCA1,IGF1,RDX,SIPA1,NRP1,CYTH4
	GO Biological Processes	GO:0042176	regulation of protein catabolic process	-12.07824471	-8.031	33/364	CD81,CEBPA,DAB2,HSPA1A,HSPA1B,HSP90AA1,HSP90AB1,LRP1,SMAD4,MDM4,NEDD4,RDX,SNRNP70,TIMP2,TMF1,TNF,XPO1,USP9X,F8A1,ADAM9,IER3,HECTD1,SNX9,FBXO11,FBXL20,TMEM259,RNF217,ZFP36L1,IGF1,PIK3C2A,ROCK2,KCNQ1OT1,SESN1
	GO Biological Processes	GO:0001819	positive regulation of cytokine production	-11.07612604	-7.205	70/497	RUNX1,CD4,CD14,CD81,HGF,HLA-A,HSPA1A,HSPA1B,HSP90AA1,IFI16,IL6ST,NAIP,PIK3R1,PTAFR,STAT1,TLR4,TMF1,TNF,CD84,ROCK2,TMED10,APPL1,TLR7,RBM47,CLEC7A,UNC93B1,RNF135,CARD11,TMEM106A,LACC1,SERPING1,C1QB,C2,CMKLR1,FCGR1A,FPR3,IKBKB,INPP5D,IPO5,NAGLU,NFATC2,STX7,RIF1,CALR,CEBPA,GAS6,CXCL10,TNFRSF1A,VEGFB,NRP1,A2M,IGF1,IER3,NR1D2,ABHD12,ASH1L,SLAMF8,ADORA3,RBPJ,JUN,LRP1,SIGLEC1,CD163,STAB1,PRDM1,MRC1,NEDD4,IPO7,SENP7,TUBB
	GO Biological Processes	GO:0030162	regulation of proteolysis	-10.21801838	-6.666	37/719	A2M,ASPH,SERPING1,RUNX1,CEBPA,CTSB,CTSD,CTSZ,DAB2,GAS6,HGF,HSPA1A,HSPA1B,HSP90AB1,IFI16,LRP1,NAIP,PMAIP1,PRNP,TIMP2,TMF1,TNF,XPO1,USP9X,F8A1,ADAM9,ROCK2,TMED10,HECTD1,SNX9,GSAP,UACA,CLEC7A,TMEM259,RNF217,IKBKB,RDX
Cluster 4 FOLR2+ CKD	Reactome Gene Sets	R-HSA-2262752	Cellular responses to stress	-19.28877949	-15.240	65/789	BLVRB,CAPZB,CDKN1A,EPAS1,FOS,GPX1,GPX3,H1-0,HBA2,HBB,HIF1A,HSPA1A,HSPA1B,HSPA5,HSPA6,DNAJB1,ID1,IGFBP7,CXCL8,JUN,LMNA,MEF2C,NCF4,RB1,RHEB,RPL21,RPL27A,RPL31,RPL37A,RPL38,RPL36A,RPLP2,RPS6,RPS17,RPS18,RPS20,RPS25,RPS26,RPS27,RPS29,CCL2,ST13,UBB,UBE2E1,VEGFA,SEM1,H2AC18,H4C3,SQSTM1,HERPUD1,DNAJB6,CITED2,TUBA1B,HSPH1,RPL35,RPL13A,LY96,PPP1R15A,RPL36,SESN1,RPS27L,TSPYL2,COX20,ANAPC16,H2AC19,C1QA,C1QB,C1QC,C3AR1,F13A1,FCGR2A,IL6ST,NRP1,TRMT112,EIF4A2,EIF4E,ABL2,CXCL12,SRGAP2,UPF2,SRGAP1,HNMT,KPNA5,AP2A2,RHOB,EGR2,ITGA1,PMP22,ITSN1,IRS2,NRP2,RPS6KA5,SEMA3E,PLXND1,PSENEN,ADGRG6,MRPL14,MRPL32,MRPL55,ACTA2,U2AF1,ZFP36L1,GTF2H2,HSPB1,SRSF7,TRA2B,SNRPD2,SNRPN,ZFP36,TNFSF13,CWC25,TRMT1,LSM2,GEMIN6,LOC102724594,BCL2L1,BST2,RUNX1,CTSL,ST6GAL1,YWHAH,ATP1B1,FXYD2,SEC23A,CYSLTR1,MGAT4A,ISCU,BLNK,ABCA1,APOE,IGF1,MAN1A1,TMEM59,WDR45,PIGN,DPM3,CHST12,POMGNT1,GAL3ST4,B3GNT5,TMEM106A,KRTCAP2,GGTA1,MGST2,P2RX7,TECR,DIP2A,ACSL6,SGMS1,BCAT1,GATM,DUOX2,PPM1K,HPGDS,RRP7BP
	KEGG Pathway	hsa05323	Rheumatoid arthritis	-17.38425425	-13.512	23/93	CTSL,FOS,CXCL2,CXCL3,HLA-DMA,HLA-DMB,HLA-DOA,HLA-DPA1,HLA-DPB1,HLA-DQA1,HLA-DQA2,HLA-DQB1,HLA-DRA,HLA-DRB1,CXCL8,JUN,CCL2,CCL3,CCL3L1,CCL5,CXCL12,VEGFA,TNFSF13,ABL2,ACTA2,APOE,AXL,PRDM1,ZFP36L1,ZFP36L2,RUNX1,CD74,CD81,CDKN1A,CTSC,GPR183,EGR3,GAS6,ID2,IGF1,IL6ST,IL10,KLRD1,LAMP1,LDLR,MEF2C,PDGFB,NECTIN2,STX4,IRS2,CD84,CD83,HSPH1,LILRB4,VSIG4,IKZF3,TNFRSF21,CD209,PELI1,SLC39A10,CD276,SIRPA,MILR1,CTSD,LGMN,FCGR1A,CTSB,HSPA1A,HSPA1B,HSPA5,HSPA6,C1QA,C1QB,C1QC,C3AR1,DEFB1,FCGR2A,FPR3,KRT10,ARL2,ZFHX3,DAB2,EMP2,CCN1,TPM1,ADAM9,NRP1,CITED2,RIN2,FMN1,AP2A2,AP1B1,CAPZB,TUBA1B,SEC23A,OSBPL1A,RHOB,BST2,EPS15,GSN,ITGA1,NPC2,LTBR,CD14,ITGB5,MRC1,MSR1,NCF4,COLEC12,ITPR2,LAIR1,CD99,SIGLEC1,UBB,UBE2E1,SEM1,IFITM1,RNF14,CLEC2B,CD96,HCST,LILRB5,WWP1,LY96,MYLIP,BLNK,SERPING1,IGHG1,P2RX7,RAB3B,H2AC18,H4C3,H2AC19,BCL2L1,CSF1R,ST6GAL1,KLF4,CCND1,EGR1,EGR2,RB1,TGFBR1,ZFP36,ANAPC16,MAF,HIF1A,CD59,RBPJ,RPS6,EIF4A2,EIF4E,IFI27,KPNA5,MT2A,GADD45B,DNAJB1,HNRNPUL1,PTPRF,RHEB,SRSF7,TSC2,ZNF669,ZNF556,ZNF527,ZNF605
	GO Biological Processes	GO:0044403	biological process involved in symbiotic interaction	-14.27077989	-11.131	32/264	APOE,AXL,BCL2L1,CD81,CSF1R,CTSB,CTSL,STOM,EPS15,GAS6,GPX1,HSPA1A,HSPA1B,IFI27,ITGB5,JUN,LAMP1,LDLR,MRC1,NECTIN2,CCL3,CCL4,CCL5,CCL8,SIGLEC1,APOL1,NRP1,XPR1,WWP1,CD209,NUCKS1,ROMO1,NFIA,RAB1A,CCL2,ST6GAL1,MGAT4A
	GO Biological Processes	GO:0006954	inflammatory response	-14.24991409	-11.131	46/541	A2M,ALOX5AP,AXL,C1QA,C3AR1,CD14,CD68,CRHBP,CSF1R,FCGR1A,FOLR2,FOS,FPR3,CXCL2,CXCL3,HIF1A,HLA-DRB1,IFI16,IGFBP4,IGHG1,RBPJ,CXCL8,IL10,JUN,MT1X,NINJ1,P2RX7,CCL2,CCL3,CCL3L1,CCL4,CCL5,CCL8,SIGLEC1,UMOD,RPS6KA5,CD163,CD96,CYSLTR1,STAB1,LY96,PLD3,BLNK,WDR83,PLD4,NRROS,ABCA1,DEFB1,GPX1,NR4A1,IGHA1,MEF2C,MRC1,RAB1A,RGS1,RNASE1,RNASE4,RNASE6,ST13,TF,UPK1B,ZFP36,ADAM9,TRIB1,PLAAT3,IKZF3,RPL13A,PELI1,COLEC12,ROMO1,SIRPA,SLC9A9,ABL2,ZFP36L1,ZFP36L2,EIF4E,HSPA1A,HSPA1B,ID3,KCNMB1,LDLR,PDK4,PLAT,SGK1,YWHAH,RNF14,HSPA5
	GO Biological Processes	GO:0006935	chemotaxis	-13.52317134	-10.496	43/500	C3AR1,CSF1R,DEFB1,GPR183,EGR2,EGR3,FPR3,GAS6,CXCL2,CXCL3,NR4A1,CCN1,CXCL8,IL10,ITGA1,NINJ1,PDGFB,PLAU,RAB13,CCL2,CCL3,CCL3L1,CCL4,CCL5,CCL8,CXCL12,TRPM2,TSC2,VEGFA,KLF7,NRP2,NRP1,CH25H,RPS6KA5,FEZ2,SEMA3E,SEMA6B,CYSLTR1,PLXND1,RPL13A,CXCL16,EMB,HMCN2,BCL2L1,STMN1,MEF2A,SLC11A2,NR4A2,NECTIN2,SGK1,UBB,STK25,DIP2A,SRGAP2,RAPH1,SLIRP,PNPT1,C12orf57,PMP22,TPM1,MEF2C,RB1,SLC1A3,APOE,JUN,PTPRF,BTG2,TNFRSF21,CAPZB,P2RX7,AXL,HIF1A,DNAJB1,ID2,RBPJ,TRA2B,SYNE2,UQCRQ,SRGAP2C,ZFHX3,HSPA5,ID1,SELENOP,YWHAH,FZD1,IRS2,C2CD3,WLS,CDK5RAP3,B3GNT5

Supplementary Table S4: Functional enrichment results of the 2 FOLR2+ clusters of myeloid cells identified in **Fig. 3** characterized by the Metascape tool. The p-value is the result of a hypergeometric enrichment test. Q-value correspond to the Bonferonni corrected p-value (or False Discovery Rate).

	Pt #1	Pt #2
Age	53	66
Gender	M	M
Serum creatinine at Nx (µM)	114	195
eGFR at Nx (mL/min/1.73m2)	62	30
HTN	Yes	Yes
Diabetes mellitus	Yes	Yes
Cause of CKD	Vascular and diabetic nephropathy	Vascular and diabetic nephropathy

Supplementary Table S5: Clinical and biological data of patients analyzed by spatial transcriptomics. Abbreviations: CKD, chronic kidney disease; Nx, nephrectomy; eGFR, estimated glomerular filtration rate; HTN, arterial hypertension.

Cell
GTTATGGCAGTCTTCC-CDm1
AGGACTTCATCCGGCA-CDm1
ATTGGGTGTTGGAGAC-CDm1
ACGTCCTAGCTATCCA-CDm2
TTGTTGTAGGTAAGTT-CDm2
CTGTGGGCAAGACAAT-CDm2
CGTCAAAAGATGGGCT-CDm3
TGTTCATTCTGCTAGA-CDm5
TTGCTGCTCGTTGTAG-CDm5
AGCTCAAAGTACCGGA-CDm5
TTTCCTCAGTAGACCG-CDm5
ACCGTTCGTTTGTGGT-CDm5
GTTTACTTCTGGGCGT-CDm5
TTCCAATCACCGGTCA-CDm5
ATACCTTGTGTGTTTG-CDm5
ATTTCTGTCATTACTC-CDm5
AGAGTGGTCCGTAGGC-CDm8
GCGAGAAAGAGTACAT-CDm8
CTGCCTACAAACCCAT-CDm8
ACGGCCATCGTTTATC-CDm8
CAGCAGCTCGGATGGA-CDm8
TAGACCACATAGTAAG-CDm8
TGTCCCACATCTACGA-CDm8
GACTACAGTCTAAAGA-CDm8
TTTCCTCTCTTAGCCC-CDm8
ATTGGACGTCTGGTCG-CDm8
CTTCTCTCACAGGAGT-CDm8
AGGGAGTGTTCTGAAC-CDm8
CATCAAGGTCTGCAAT-CDm8
GAGCAGAGTTTGACTG-CDm8
CTAGCCTGTCCGCTGA-CDm8
CGGACGTAGAGGTTGC-CDm8
GACCAATGTGGTCTCG-CDm8
CAAGGCCAGTCCAGGA-CDm8
CAGTAACTCAGCAACT-CDm8
CATCCACCAACACCTA-CDm8
TTCTTAGAGGATCGCA-CDm8
GCATACAGTCGCGAAA-CDm8
TACTCATGTGACAAAT-CDm8
TTGCCGTGTCTCACCT-CDm8
GACGTGCTCCATGAGT-CDm8
GTGTGCGCATTTGCCC-CDm8
TACGGATCACCGATAT-CDm8
ACTGAACGTTCTGAAC-CDm8
GGATGTTCATCGACGC-CDm8
TCACAAGCAAGCCGTC-CDm8
CACACAATCCTTTCTC-CDm8
GTGCAGCCATTGAGCT-CDm8
TGAGAGGTCTTAACCT-CDm8
CCACGGAGTGGGTATG-CDm8
CAGAGAGTCGTACCGG-CDm8
CATCAGACACGAAACG-CDm8
CACACAAAGGGCATGT-CDm8
TCATTACAGTGTACCT-CDm8
TCTCTAATCGCGATCG-CDm8
CGAACATCAGCCTTGG-CDm8
AGCCTAAAGAAAGTGG-CDm8
GGGAGATCAACAACCT-CDm8
CGATTGAAGCTGGAAC-CDm8
CTGAAGTCATCGATTG-CDm8
TGATTTCAGGAATTAC-CDm8
AGGGAGTGTGCAGACA-CDm8
TAGACCATCTCACATT-CDm8
GTGTTAGCAGTCAGAG-CDm8
AACTGGTGTGCCTGCA-CDm8
CATCGGGAGTACATGA-CDm8
AAGTCTGAGCCAGTTT-CDm8
ACTGAACGTAGCGTCC-CDm8
GCAATCACACGTGAGA-CDm8
CAGCTAACAATGGATA-CDm8
CGGGTCATCGTCACGG-CDm8
AAAGCAATCCTTTCTC-CDm8
GGTGTTAAGAGGTACC-CDm8
CAAGTTGAGCACGCCT-CDm8
TGTGTTTCACGAAACG-CDm8
ATCTACTGTTTAAGCC-CDm8
TCTATTGCATTCCTGC-CDm8
TAAGAGATCATGCAAC-CDm8
CTAGAGTGTTCAGTAC-CDm8
AACTGGTAGGCGACAT-CDm8
GCGAGAACAATGTTGC-CDm8
AGGGTGACAGGCTGAA-CDm8
CTAGTGAAGTCATCCA-CDm8
CCTACCAAGTGATCGG-CDm8
CTAAGACAGTGGGATC-CDm8
GGACAGACAATCGGTT-CDm8
GATGAAACAAGCCGTC-CDm8
ATAACGCTCTGGTATG-CDm8
TTTGGTTGTAAGAGGA-CDm8
TACCTATAGTACCGGA-CDm8
GGTGCGTTCTTGGGTA-CDm8
TTTCCTCAGGCAGGTT-CDm8
CCTAAAGCAGTAAGCG-CDm8
TGTATTCAGATGTTAG-CDm8
CTGTGCTCACATGACT-CDm8
CTCGTACCAAGACGTG-CDm8
CTTACCGTCGAGAACG-CDm8
GGACATTGTCTCTCGT-CDm8
GTGCGGTGTCTAGAGG-CDm8
AGCTTGACAAGGCTCC-CDm8

Cell (continued 1)
CTAGCCTAGAAGGTTT-CDm8
TTTCCTCGTACCGGCT-CDm8
ACGGGTCCACTTCGAA-CDm8
GGAACTTGTTTAAGCC-CDm8
TCAGATGAGGCCATAG-CDm8
ACTGAGTTCTGCTGCT-CDm8
GTACTTTCAAGCGTAG-CDm8
CCTCTGAAGGTGCACA-CDm8
GATGCTAAGGCGACAT-CDm8
TGAGCATGTTTGCATG-CDm8
TCTCTAAAGTTTCCTT-CDm8
AGCGTATAGTAGGCCA-CDm8
TGCGCAGGTACCGTAT-CDm8
CGCGTTTGTGCAACGA-CDm8
GACCTGGTCATCGCTC-CDm8
GTCTCGTCAGTCAGCC-CDm8
CAGAGAGCACTTGGAT-CDm8
CATTCGCCATTCCTCG-CDm8
TTTACTGGTGTTCTTT-CDm8
GGGTTGCTCTGTCTAT-CDm8
CATCGGGGTGCCTTGG-CDm8
CTCTACGAGAGGTTAT-CDm8
CCTCTGACAGGTCTCG-CDm8
GCGCAGTCAGGGTACA-CDm8
TCTGAGATCGAGGTAG-CDm8
AGAGCTTAGTGTGAAT-CDm8
GCTGCTTTCAAACGGG-CDm8
CCACCTACAAGTAGTA-CDm8
CTGATAGTCTCCAACC-CDm8
GCGCGATGTCGACTAT-CDm8
CTCGGAGTCTTAGAGC-CDm8
GTAGTCATCTTGTATC-CDm8
CAGATCAAGCCGATTT-CDm8
AGGCCGTGTAAACCTC-CDm8
GCGACCATCTTCAACT-CDm8
CATTATCAGATATACG-CDm8
TCTTAGTCACGCCACA-CDm9
GAACACTTCTAGGCCG-CDm9
AGACTCAGTGCACATT-CDm9
ATGCATGGTATCATGC-CDm9
ATTCTACTCTCCATAT-CDm9
TACACCCCACCGCTGA-CDm9
GGGTGAACAACACTAC-CDm9
GTTACAGGTGTGTCGC-CDm9
ATTGGGTCACATTCTT-CDm9
GAGTCTACACATAACC-CDm9
TACTTGTGTCATGGCC-CDm9
CACAGATCAATCAGCT-CDm9
TCGGGACAGCTGTGCC-CDm9
GGAATCTGTACTTCCC-CDm9
CCTACGTGTTTACTTC-CDm9
CACAACAAGGCGAAGG-CDm9
GCAGGCTGTGACACGA-CDm9
CTACCCATCATCGTAG-CDm9
CAGATTGCAGCAGTCC-CDm9
TCCTTTCTCTCTATAC-CDm9
ACTATTCCAATAACCC-CDm9
TAAGCCACACGGCCAT-CDm9
ACTCTCGTCGGTCAGC-CDm9
ACAACCAGTATCGGTT-CDm9
GCCCAGAAGTAGTCCT-CDm9
CCCAACTGTCGAACGA-CDm9
AGTACCATCCATCGTC-CDm9
AAAGAACCACACACTA-CDm9
TGACTCCCATCCGCGA-CDm9
GAAGCCCCACTGTCGG-CDm9
AGCCACGAGACTCATC-CDm9
ACATTTCTCCTACTGC-CDm9
AACAAGACATTCAGCA-CDm9
AATCGTGGTTACGTAC-CDm9
ACGTAACGTCAGGTGA-CDm9
GTCAGCGCACTACGGC-CDm9
GCAACCGTCAGCTAGT-CDm9
TGTTCCGCAGGAAGTC-CDm9
CTTCCTTGTACGAGTG-CDm9
TCCACGTTCATAGCAC-CDm9
TCTGTCGCAGCACCCA-CDm9
ACTATTCTCGGATTAC-CDm9
ATGCCTCCACAATGTC-CDm9
AAGTCGTCAACTGGTT-CDm9
CCGAACGGTGAGCTCC-CDm9
CTTCCGAGTCTAGGCC-CDm9
TGTGAGTGTCATCCGG-CDm9
GATGAGGTCTCGCGTT-CDm9
GAGAGGTCAATGAAAC-CDm9
CGATGCGTCACTTGTT-CDm9
TGACCCTTCATGGAGG-CDm9
GCCGTGACACGCCACA-CDm9
AACCAACGTTCGAACT-CDm9
GTTACGAGTCGAATGG-CDm9
AGTACCATCGTCGATA-CDm9
CCGATCTGTTCTTGCC-CDm9
CCAATGAGTCCCGGTA-CDm9
ACCTGTCAGAGAGCCT-CDm9
GTGGTTACATCAGTGT-CDm9
TACTTCAAGTGGAATT-CDm9
CGAGGAACATGAGGGT-CDm9
TCGGGCAGTCGGCCTA-CDm9
CAGCAATAGGAAAGGT-CDm9
CATTCCGCATTGCTGA-CDm9

Cell (continued 2)
CTCTGGTAGTTCCGTA-CDm9
AATTTCCAGTACTCGT-CDm9
CGGAATTAGCCTTTGA-CDm9
TCAATTCCACCTGCAG-CDm9
TTCTAGTTCGCATAGT-CDm9
TCACGCTGTTATAGAG-CDm9
GGTGTTACAGTGAGCA-CDm9
TCCGGGATCCTATTTG-CDm9
TACGCTCAGGGCTTCC-CDm9
AACTTCTTCCCATAAG-CDm9
GGGACTCGTATGAGGC-CDm9
CTTCCTTAGCATGAAT-CDm9
TTAGGGTTCTCTGACC-CDm9
ATTCGTTAGTGAGGTC-CDm9
GGCTGTGCAACGGGTA-CDm9
CGTAGTATCTTAGCTT-CDm9
AAACGCTCAGTAGTGG-CDm9
GATGAGGGTACAAACA-CDm9
AAGGAATGTGACTCTA-CDm9
GACGTTATCTTGTTAC-CDm9
GAGTTTGTCGCCTTGT-CDm9
GGCTTTCAGTGCCAGA-CDm9
AGATGAAGTGTTAGCT-CDm9
TACATTCGTGTCGCTG-CDm9
GAAGGACGTCAAGGCA-CDm9
TGGCGTGAGCGTTCAT-CDm9
TCAGTCCCAAGGCCTC-CDm9
AAGGTAATCAGACATC-CDm9
ATTACCTTCTCAGAAC-CDm9
GTCAAGTAGGTGGGTT-CDm9
GATGTTGTCGCAGTCG-CDm9
TCCACCATCACCTCTG-CDm9
ACATCCCAGCCATTTG-CDm9
TCTTGCGTCTCCCAAC-CDm9
TACACCCCAACGTATC-CDm9
TCATACTGTCTCAGAT-CDm9
TAAGCGTCACTGCGAC-CDm9
CGGGACTGTAGGGTAC-CDm9
TGGGATTAGCGACTAG-CDm9
AGAAGCGAGGATACGC-CDm9
TTAGGGTCAAAGCGTG-CDm9
AGATGAACAATTGAAG-CDm9
TAACCAGGTTTGGGAG-CDm9
TAAGCGTGTCTACTGA-CDm9
CTGGCAGTCGAGAGCA-CDm9
AATGAAGTCGTAACAC-CDm9
GAGCTGCAGCATGCGA-CDm9
CCTCCAAAGGGAGGAC-CDm9
ACCAACAGTACCACGC-CDm9
AGCCAATGTATCAAGA-CDm9
AGGAGGTGTTCGTAAC-CDm9
GGTAATCAGTTACGTC-CDm9
TCATGCCAGTTTCTTC-CDm9
AGGACGAAGGGACACT-CDm9
AACCTTTAGAGAGGTA-CDm9
AGGGTCCCAGCTTTGA-CDm9
CATGCCTAGGAGATAG-CDm9
GGGCGTTAGCGGTAGT-CDm9
TGGAGAGAGATACCAA-CDm9
AAGATAGTCTATCGCC-CDm9
TTCCAATTCTAACACG-CDm9
CAATGACTCGCTGTCT-CDm9
ACGTTCCAGGGACAGG-CDm9
GCCAGGTAGTAAGACT-CDm9
TAACGACAGGATATAC-CDm9
GTTGTCCAGGGCGAAG-CDm9
GATTTCTGTTTCGTAG-CDm9
TTTGGAGGTGTTGACT-CDm9
ATCACAGTCATCAGTG-CDm9
AGAACAAGTGCTAGCC-CDm9
TCACGGGCACTCCCTA-CDm9
GCATGATCACTGTCCT-CDm9
TGCGACGTCGTGGACC-CDm9
GAAGGACTCCTCGATC-CDm9
ACAGGGAAGCACACAG-CDm9
AGGTTACCATAACCCA-CDm9
CAAGCTACAGGTTCAT-CDm9
GGGAGTAAGTAAGACT-CDm9
AGTTCCCCAATTGCGT-CDm9
CCTCATGTCTATACGG-CDm9
TAAGCCAAGGAGAGTA-CDm9
ATGGGTTGTATAGGAT-CDm9
TATCGCCGTTAGGCTT-CDm9
ACGGAAGAGAAACACT-CDm9
GTTGTGACACGCTATA-CDm9
TTCCACGAGAAACCCG-CDm9
AACCTGATCCCGTGAG-CDm9
TAACGACTCAACTGGT-CDm9
CACTGAAAGCAATTCC-CDm9
AATAGAGGTGGCTACC-CDm9
GAAATGAGTAGGTTTC-CDm9
AGGTGTTAGAAGGATG-CDm9
CGGGTCATCTGCGAGC-CDm9
GTCTGTCTCCCGTGTT-CDm9
GTATTGGAGTAAGGGA-CDm9
CATTGCCGTTTACTTC-CDm9
TTCTGTAGTAACATGA-CDm9
AGTACTGAGATGTTAG-CDm9
AACCACAGTGAGACGT-CDm9
GACCCAGTCTTACCAT-CDm9

Cell (continued 3)
AGAACCTAGCCTGACC-CDm9
GGGTTATAGGAACATT-CDm9
CTCTCGAAGTCACAGG-CDm9
ATCACGAGTCAGACGA-CDm9
CGCCATTTCATTCGGA-CDm9
AAGTGAATCGGTCTGG-CDm9
AATGGCTGTTGTAGCT-CDm9
ATCACAGTCCCTCGTA-CDm9
AGACTCATCTTCACGC-CDm9
AGTTAGCGTACGAAAT-CDm9
TGGTTAGGTGACATCT-CDm9
TTCCGGTCAGCACAGA-CDm9
GCCAGCAAGTAGCAAT-CDm9
ATTCAGGGTTCTCAGA-CDm9
GCACGTGTCCTACACC-CDm9
GTCTGTCGTCTAGTGT-CDm9
TATCGCCCAGCTCGGT-CDm9
AAAGGATAGAAGGCTC-CDm9
AGCTTCCCACTCAAGT-CDm9
TCCGATCAGCAGCCCT-CDm9
AGTCTCCGTGGTTTAC-CDm9
TGGATGTCAACAGTGG-CDm9
ACGTTCCCAGTATTCG-CDm9
TAAGTCGAGTACCATC-CDm9
ACTATTCCACGGCCAT-CDm9
GGCGTCACATGACAGG-CDm9
ACACGCGAGACTCATC-CDm9
TTGCATTTCCAGTTCC-CDm9
CATTGTTCATCACCAA-CDm9
CATTGAGAGATCCCAT-CDm9
AGGGCTCCAGGCTATT-CDm9
CTTCTCTGTATTCCGA-CDm9
TCACTATAGCGATGCA-CDm9
GGGAGTATCACCTCAC-CDm9
CCTAACCAGCGAATGC-CDm9
TTTGATCTCCCTTGGT-CDm9
GCATGATTCTCACCCA-CDm9
AAGTCGTGTAAGGAGA-CDm9
AGGTAGGAGTGATCGG-CDm9
CAGGCCAGTACGTGAG-CDm9
TCACTATAGCGATCGA-CDm9
GCAGGCTTCGAGAAAT-CDm9
ACTTCCGCAAGAATGT-CDm9
TAGACCAAGAGGCCAT-CDm9
GTCATCCTCGGCTGAC-CDm9
GATAGAAAGACAACAT-CDm9
CTGAATGAGCGGACAT-CDm9
CTCCAACAGCTGAAGC-CDm9
ACTATTCAGTAGGGTC-CDm9
CGTTGGGGTCACAGAG-CDm9
GCCATTCCATTCTCCG-CDm9
GCTCAAATCAGCGCAC-CDm9
TAATCTCTCCCTCATG-CDm9
AGACAAAAGTAGCTCT-CDm9
TCGTGCTAGTGTAGAT-CDm9
TGGAGGACAGTGCCTG-CDm9
TTACAGGTCTTGAACG-CDm9
TACGTCCAGGCAGCTA-CDm9
CTTTCGGCAAGTCGTT-CDm9
CTCCCAATCCGTAGTA-CDm9
CGATCGGTCACCCTTG-CDm9
CTGCCATTCGCTCATC-CDm9
AGGTCTACACTGTTCC-CDm9
TACAACGTCCGACATA-CDm9
CAGCAGCTCGGAATTC-CDm9
TTTATGCTCCTAAACG-CDm9
GGTGATTAGCCTTCTC-CDm9
CCAAGCGTCCGTATAG-CDm9
GAAATGAAGGAGTACC-CDm10
TACCTTAGTTCGTGAT-CDm10
ATTACTCCAGCTTAAC-CDm10
CCTTTCTTCACGACTA-CDm10
ATGCGATAGAATGTGT-CDm10
GTGGGTCGTTAAGACA-CDm10
TCTGAGAAGCACCGCT-CDm10
TTTGGTTAGGAGTACC-CDm10
TGCGTGGCACTCTGTC-CDm10
TATCAGGAGCAATATG-CDm10
CTCGGAGCAGTCAGCC-CDm10
CCGGTAGGTTATGCGT-CDm10
CAAGAAATCGCTAGCG-CDm11
GTAGTCACAGATCGGA-CDm11
CATCAAGTCGGCTACG-CDm13
CGTAATGGTAGCGCTC-CDp1
CCCAACTTCCAAGCAT-CDp1
AACTTCTCACACCTAA-CDp1
CCCATTGAGCGCCCAT-CDp1
TCTATCACAACTTGCA-CDp1
CTAAGTGGTCAGGCAA-CDp1
CGATGGCTCGGCTACG-CDp2

Supplementary Table S6: List of cells at the node of differentiation from CXCL-iFibro to ECM-myofibro used for transcription factor inference.

TF	Viper score	SD	Viper Score	SD
SIX2	-0.163542462900246	0.944348745447616	0.869019753842495	0.826616726962196
TCF4	-0.120260513944157	0.963257154317185	0.639031358409149	0.951985080142323
HOXB13	-0.110437224435607	0.967806503116646	0.586833094549989	0.96747178552879
MITF	-0.109415000496486	0.968227289028611	0.581401277147992	0.969150395690844
MEIS2	-0.108902681851929	0.966280211385119	0.578678956507307	0.981436614565266
TCF12	-0.102694425808802	0.97486410059232	0.545689988121274	0.958370678037717
MAFK	-0.0958523865830463	0.958909559259757	0.509333269882454	1.06290195898468
MAFF	-0.0936999137323328	0.955313056162432	0.49789562002867	1.08649016962359
KLF3	-0.091564902849794	0.96936386747074	0.486550758280272	1.0242103988626
SOX13	-0.0913754961787445	1.00141180918048	0.485544303224305	0.843725189276299
FOXJ2	-0.0911183699061039	0.972572019819058	0.484178004795178	1.00915809759841
KLF9	-0.0901997615991035	0.984011582811971	0.479296772418765	0.950869696122149
GABPA	-0.0887853301166295	0.972326227952024	0.471780871796217	1.01743870428906
MEIS1	-0.0878192321241408	0.987471508435431	0.466647292267501	0.939134845202004
ETV4	-0.0873665604080531	1.01619724961359	0.464241919031027	0.759184728305516
MEF2A	-0.0869978697767603	0.990606131792736	0.462282798225543	0.923919109201923
LHX2	-0.0864710112083952	0.957997142933275	0.45948321642109	1.09411225121073
RUNX2	-0.0859368590893831	0.987597893345258	0.456644878690652	0.9443087232582
MAFB	-0.0856429052300591	0.967359404434333	0.455082888575413	1.05135971689569
CEBPA	-0.0832269024855648	0.999145645313723	0.442244913207611	0.885641897633955
TEAD1	-0.0807629501615855	0.997126326834382	0.429152146937055	0.905342058893045
CEBPG	-0.0795525891049618	0.986180695635744	0.422720620538129	0.970823902742485
ARNT	-0.0792530798873943	0.995975638512632	0.421129110774205	0.916577770018406
NFATC1	-0.0748854242326654	0.97264515288825	0.397920587589254	1.05306209728473
PBX3	-0.0740980003094264	0.977576005151786	0.39373643301676	1.03024545461326
ESR1	-0.0739195579640351	0.977213975519964	0.392788239377522	1.03251697486596
ARID2	-0.0735222899555706	1.00235575078676	0.390677266234501	0.895257856259619
JUN	-0.0733741674484946	0.994119578797774	0.389890183892982	0.943557842155073
HOXA9	-0.073357095318664	0.999749627971152	0.389799467281518	0.911184487097916
NFE2L1	-0.073310996521093	0.986492754996456	0.389554510925811	0.985661453824629
PRDM1	-0.0730773721049346	0.941850598805428	0.388313094910538	1.19741312032898
ESRRA	-0.0728714845334319	0.995711858266964	0.387219064873718	0.935853115677112
JUNB	-0.0718206862663138	0.998087614946859	0.381635411336691	0.924986603229043
TGIF2	-0.0706707566973897	0.9912373736573	0.375525001274359	0.966512256717476
NCOA3	-0.0688615774479229	0.98879849943511	0.365911519380143	0.984157261099483
SOX11	-0.0685315985437327	0.976738587927303	0.36415810206572	1.04741187609111
POU5F1	-0.0681913721942916	0.982728125702526	0.362350232640255	1.01773423059704
FOSL2	-0.0681512282072268	0.980817662146438	0.362136918512909	1.02764873062776
ONECUT1	-0.0676135222328685	0.976670392624113	0.359279696570735	1.04977145109307
ZNF384	-0.0671494861253734	1.00502157696035	0.356813936077963	0.896212348245256
MEF2B	-0.0670746798401392	0.989011088315442	0.356416436013288	0.987189608024448
TBP	-0.0666638645821236	0.980269815921967	0.354233476505016	1.03373624912649
KLF5	-0.0642820461194688	0.982353384962297	0.341577147026979	1.02823833512649
TFAP2A	-0.064271901731534	0.994557640550723	0.341523242534244	0.963371681155145
NCOA1	-0.0641360624093296	0.985146237639713	0.340801429665269	1.01413804578289
POU4F2	-0.0641310278071391	0.992176648686339	0.34077467717126	0.976747634030267
SP3	-0.0635272390648495	0.982712638708606	0.337566309540671	1.02798893974239
CREB3L1	-0.0630405404154845	0.997692211762581	0.334980126521492	0.948655046930073
NR4A1	-0.0622421864478584	0.998022706100167	0.330737892693515	0.948579081843582
ETV1	-0.0622194984908785	0.989918864304548	0.330617335118183	0.993070961202319
DUX4	-0.0613244664153525	0.973909930135347	0.325861380363953	1.07626035755345
NCOA2	-0.0610463882834937	0.980636902913533	0.324383749506407	1.04358128765847
ZNF740	-0.0607734003877793	0.991124789619862	0.322933166766419	0.989656318834099
SP1	-0.0575554834212657	1.00466241914825	0.305834039356122	0.920651619261352
E2F7	-0.0569143359348652	1.01535876182458	0.302427157614679	0.856942472193268
FOXK2	-0.0567278389265518	0.998717300813154	0.301436163707744	0.956347760521309
ERG	-0.0565216879129834	1.01785362604945	0.300340733812128	0.84183638845807
MAX	-0.0563118074894716	1.00662779077239	0.299225486855811	0.911714802046844
TCF7	-0.0559041861552396	0.978014577356765	0.297059498981761	1.06627837191104
MYBL2	-0.0549975968633517	0.997408039448898	0.292242132352317	0.967031298139681
TEAD2	-0.0544006825288092	0.990274475554523	0.289070293437399	1.00663416435582
ATF6	-0.0535019227762845	0.993438350820754	0.284294530830847	0.991453457159592
FOSL1	-0.0533436013119511	0.975726600900879	0.283453254030168	1.08183705591533
CTCF	-0.0526719008015239	0.975714574655406	0.279884021906146	1.08300962727403
GATA3	-0.0526507283451858	0.985780221277688	0.279771517285205	1.03306799198131
PAX6	-0.0525050858668871	1.00299095592666	0.278997613135816	0.940429241050192
RBPJ	-0.0524860482508488	0.998885416742801	0.278896452470197	0.963586143283566
ZFX	-0.0522104416786461	1.00838168516625	0.277431954802228	0.90960098887655
HIF1A	-0.0520627088413564	1.00710213937171	0.276646943058971	0.917447340471298
ATF7	-0.0519951000027408	0.992754640723073	0.276287688249863	0.997817561880274
ELF1	-0.0515812283630373	1.01977228488411	0.274088487968307	0.840144558544895
AR	-0.0515020440565898	1.00563155400927	0.273667724300691	0.927109193257405
OTX2	-0.0511030186845514	1.00501323676462	0.271547413010072	0.931438588886746
YY1	-0.0504074283103348	0.990681271366468	0.267851236707857	1.01152171899141
EBF1	-0.0501241336290142	0.9894941205696	0.266345886538498	1.01820079957697
IRF2	-0.0490218746794923	1.00513603189895	0.260488785061605	0.934513633539224
PBX2	-0.0485204556558003	0.991686343697111	0.257824382014176	1.00937045718968
NR2C2	-0.0472082485818802	1.00700878577384	0.250851673837045	0.92684655133756
SOX6	-0.0469278001990966	0.984027253990419	0.24936144811676	1.0512058552788
XBP1	-0.0467820963376691	0.959569206791589	0.248587217794282	1.16624309850949
GFI1B	-0.0465936896955106	1.00059356856582	0.247586076617332	0.964356950676934
SMAD5	-0.0457692167772926	0.986712271760346	0.243205053855801	1.03938881827229
CUX1	-0.0457209208610866	1.02551100573866	0.242948422614788	0.813813679062296
IRF4	-0.044202655544806	1.01257215981726	0.234880777502789	0.898982797043877
TP53	-0.0439947723345718	1.00170621407386	0.233776143189604	0.962303381857819
ATF4	-0.0438737164240807	1.00395268197543	0.233132885312268	0.94987050787989
IRF9	-0.0431396733580693	0.964590490282563	0.229232381961507	1.14868087565836
GRHL2	-0.0429484748777882	0.994031431361945	0.228216405723149	1.00556138103819
THAP1	-0.0428226272319078	0.986996256394072	0.227547685879358	1.04219546792839
GATA6	-0.0426102645206814	0.980053954951668	0.22641924872755	1.07700176877528
NFE2L2	-0.0423094283940867	0.979759729176919	0.224820688133283	1.0788359693398
HNF4G	-0.0422039660760826	0.985942877198134	0.224260290325852	1.04837287390586
ZBTB33	-0.0420112004788339	0.982716190171237	0.22323598685812	1.06473522977697
MYOD1	-0.0419651850641331	0.977929612957782	0.222991473576082	1.08814567894988
ASCL1	-0.0414780859630511	0.970245792751243	0.220403162666408	1.12499818937969
RUNX1	-0.0414715426921419	1.01057121376326	0.220368393520991	0.915304215793898
PDX1	-0.0410163958842946	0.995908806945752	0.217949868326352	0.998318219753981
EGR1	-0.0405729873526279	1.01959236838682	0.215593717109057	0.861498461946876
KLF6	-0.0396957928802431	1.02518914640083	0.210932546481293	0.826598565860068
CLOCK	-0.038847857069518	0.995792325595114	0.206426848349796	1.00187316912295
ELF5	-0.0382424607971517	0.968186372400472	0.20320993874566	1.13830737397606
USF1	-0.0373776587099076	1.0033977612896	0.198614617850685	0.962342860741204
MAZ	-0.0361235869179683	1.00098152512479	0.191950824603345	0.977327145797411
NFATC2	-0.0358573727538552	0.988265015399018	0.19053623561362	1.04466045144301
CREB1	-0.0352015838713311	0.98965701249543	0.187051553512375	1.03833841304445
HINFP	-0.0348719225437553	0.985424366977874	0.18529982371289	1.06005102544113
NRF1	-0.0340793913942973	0.995897630582679	0.181088530742247	1.00717861891953
STAT2	-0.0332488598648378	0.988638210991248	0.17667531418375	1.04569177019312
PPARG	-0.0331816062950676	0.992214015332511	0.17631794717575	1.0274639979004
CREM	-0.0326415059066453	0.997412421992285	0.173448001974531	1.00074261605617
SP2	-0.0325388519143888	0.965661072160484	0.172902526839207	1.15567312382301
SP4	-0.0321681904251569	1.01012807452328	0.170932933435637	0.930478575335891
BACH1	-0.0315957497829762	0.984644126344544	0.167891141003666	1.06738921615839
FOXO4	-0.0307781227414962	0.991232700385441	0.163546495351878	1.03504417411336
FOXO3	-0.0307259532218768	1.01233103233115	0.163269280845654	0.91923810708776
CEBPD	-0.0304007673435415	0.966761942629753	0.161541332354895	1.15271194190434
MAFG	-0.0300565904759486	0.995537625326496	0.159712470960443	1.01340544912411
E2F1	-0.0293256620079924	0.988393417369455	0.155828517728754	1.05089546004457
E2F4	-0.0269496647083103	1.00483003448083	0.143203120312778	0.966139135700078
JUND	-0.0261234469160797	1.00960615785533	0.138812825769761	0.93988042797867
RFX1	-0.0252556143454622	1.01394926879546	0.134201401718049	0.915320038334041
ARNTL	-0.0251616066610406	0.989011394610198	0.133701870689064	1.05143857360904
HNF4A	-0.024736150301304	0.973335201119643	0.13144111238536	1.12739420757856
SMAD3	-0.0246665092085287	0.99996304786807	0.131071058735508	0.994827649636812
AHR	-0.0243277770766027	1.02191673440018	0.129271129171762	0.867458593010596
ATF2	-0.0241181516043703	1.02004361944347	0.128157236956556	0.87938383647113
ZBTB11	-0.0240124170536669	0.977166414703634	0.127595392579305	1.11003854178833
PAX8	-0.0235899243213883	0.96537744495327	0.125350382178358	1.16427338681881
POU2F1	-0.0235317939221323	1.00964520225707	0.125041493194075	0.941972986039569
RFX2	-0.0229189882060846	0.985842953628613	0.12178521184017	1.06896807398642
STAT4	-0.022873546055553	1.01164698311568	0.121543744726566	0.930953777664868
ZNF217	-0.0227252983116561	0.996556179976965	0.120755996910957	1.0145111583059
HSF1	-0.02258145863156	0.999501652427152	0.11999167233633	0.99898019137127
NFYB	-0.0224407652281378	0.992370746791289	0.119244066212275	1.03646421564169
STAT5B	-0.0220497892067787	1.01938597548955	0.117166526961513	0.885288372936359
NANOG	-0.0216625196983631	0.980384653985969	0.115108683103066	1.09639285184662

TF	Viper score	SD	Viper Score	SD
FOXM1	-0.0213647712553189	1.02520021741312	0.113526529611598	0.849156836218519
MYB	-0.0202801749749762	0.973159926703308	0.107763282710168	1.13121241130784
MYC	-0.0198644735771892	1.01559236740048	0.105554359596431	0.910049514714313
PROX1	-0.0198193526971858	0.979736902293347	0.105314599626223	1.10066739772979
PRDM14	-0.0193385371828906	1.0188808168661	0.102759677971822	0.890535086446838
PKNOX1	-0.0181046803356717	1.00443907966644	0.0962033013915245	0.975261229447564
TFAP4	-0.0171065948002823	0.980881936633051	0.0908997488406966	1.09673953524597
NR3C1	-0.0170367369511914	1.00880671108457	0.0905285434073136	0.951475827959735
ZKSCAN1	-0.0154032107980862	1.0012161586989	0.0818484338486572	0.994397209598144
NR1H2	-0.0151626005470793	1.00322628227025	0.0805698970246802	0.983612201757609
NEUROD1	-0.0150343005081781	0.984999626847886	0.0798881458375633	1.07783790049498
TEAD4	-0.0143518068256211	1.02464843133854	0.0762615617596639	0.857678555849873
BHLHE40	-0.0142249155499514	1.02151291801744	0.0755872963536789	0.877553988639697
KDM5B	-0.0140732903539184	1.00567591024516	0.0747816016845432	0.970676378171937
TP73	-0.0131930345439938	0.979952074332122	0.0701041639494576	1.10300575033147
TFAP2C	-0.0127159510500714	0.99942264523111	0.0675690732268499	1.00528893184742
EPAS1	-0.0122475983999501	0.985337873016703	0.0650803758114993	1.07737671820491
STAT1	-0.0102343679310935	0.992335751253939	0.0543826217514992	1.04314123003776
BACH2	-0.00873832873755537	1.02612183181473	0.0464330801544492	0.850775564474254
TAL1	-0.00829416603074083	0.988503488167404	0.0440729214574626	1.0630253341015
NFE2	-0.00793004244845433	1.01527022913173	0.0421380686966835	0.918113999139895
PGR	-0.00777334134820763	0.993763946721238	0.0413054020659648	1.03655749214718
BATF	-0.00701197818273637	0.989686657455999	0.0372597272063066	1.05742653645104
ZNF143	-0.00636613690128041	1.02789599737986	0.0338279039264427	0.839948398174347
CEBPB	-0.00532448545693514	1.00852582016766	0.0282928540946967	0.95771345985829
CREB3	-0.00530498433815585	1.00082717953793	0.0281892305027557	1.00004116552475
IRF1	-0.00420521867806845	0.994287804041635	0.0223453776815025	1.03456322999482
SNAPC4	-0.00395538085785887	1.00652448137785	0.0210178080878451	0.969147545525212
EHF	-0.00286797495675155	0.985073849008784	0.015239631632929	1.08089355572478
CTCFL	-0.00279556267143505	0.982836154074208	0.0148548526266476	1.09175651079821
BCL6	-0.00219561436563552	1.01791992295457	0.0116668920213257	0.903351399086425
ATF1	-0.00215677816297331	1.00985817575396	0.011460527101289	0.950585849677021
HNF1A	-0.00109478406893021	0.924115735679299	0.00581738201336569	1.33863890690971
FOXP1	0.0906047347088385	0.990148010734003	-0.481448688354789	0.9147584015264
HMBOX1	0.086489577003018	0.960924372194889	-0.459581869957216	1.08022454166914
RELB	0.0840258409209123	0.995610964802223	-0.446490252736613	0.904213171751763
ESR2	0.0793970601963325	0.9923471415127	-0.421894182611885	0.937008822691663
ETS2	0.0759767679149679	0.988391610066378	-0.403719688332483	0.968482325102855
E2F6	0.074900577749725	0.995818065837642	-0.398001109219124	0.929800473949593
NFKB1	0.0738417086252478	1.00452931871967	-0.392374569361611	0.881207400343174
ZEB2	0.0736702150791029	0.99341228772679	-0.391463299734068	0.946760976105105
MNT	0.0711507020142746	0.989659327882622	-0.378075298938581	0.973952600155238
IKZF1	0.0664669359700867	0.994872800049264	-0.353187051919478	0.956558084396021
ZEB1	0.0659032090386119	0.996703633421162	-0.350191561754191	0.947629019389198
FLI1	0.0657297992961194	0.965797077009828	-0.349270109985263	1.10619353911283
VDR	0.0647476732125835	0.988818572815024	-0.344051361580588	0.993466696975392
IRF3	0.0642770496447223	0.980298570948811	-0.341550597131751	1.03870001683558
NFIC	0.0635578181431645	1.00377488628637	-0.337728798368579	0.912423641949883
E2F5	0.0608607394058033	0.983428439009432	-0.323397262332795	1.02978670694674
HBP1	0.0602498988441225	0.99686483201395	-0.320151423269756	0.95939729225559
TCF7L2	0.0600327924534038	0.992674631279649	-0.318997779507301	0.982843961085907
E2F3	0.0587072250660596	0.988608068634202	-0.311954078292201	1.00721000657731
LYL1	0.0576883170728238	0.975128910270563	-0.306539880916377	1.0771594673999
FOXL2	0.0575623156712412	1.0205558202867	-0.305870344056988	0.821682369503866
SREBF1	0.0573554068184378	0.992250059270666	-0.304770887211699	0.990527693438849
RELA	0.0573317233177382	1.01915570892066	-0.304645039590335	0.831478561311241
THAP11	0.0564091471494189	0.992917086850329	-0.299742723088066	0.988786987241597
NFKB2	0.054823636003826	0.974569055002983	-0.291317752098762	1.08491461808657
MXI1	0.0535703861418868	1.00386223165186	-0.284658326361796	0.933387709201415
FOXP2	0.053453653905127	1.00371137859437	-0.284038043299787	0.93448279418161
ATF3	0.0527958980706067	1.00669820208791	-0.280542909355582	0.918401847602204
NR5A1	0.0524267483356788	1.00036257858497	-0.278581348999396	0.955452954923876
E2F2	0.0521736296731099	0.984918746590363	-0.277236345910051	1.03827626688991
TWIST1	0.0505808125342949	0.99158819165422	-0.268772552878312	1.00645583192499
WT1	0.0488560684531403	0.969231296548044	-0.259607736290211	1.11963328134185
TFDP1	0.0483649217897768	0.943344206707887	-0.256997917745677	1.23304463888683
SREBF2	0.0479484978898213	1.01650477514284	-0.254785155453757	0.868177746822199
BHLHE22	0.0459087999959408	0.977572055494013	-0.243946760762746	1.08446526004058
ZNF639	0.0452192697643079	0.994784089762079	-0.240282786394651	0.998174428527783
MBD2	0.043272024289743	0.998297638500637	-0.229935658480792	0.982181488364553
USF2	0.0432698623841856	0.955787822506485	-0.229924170708123	1.18728017401834
TCF3	0.0432553216465759	1.00482023870065	-0.229846905220041	0.94590947478806
FOXA2	0.0427223759886693	0.9956883482319	-0.227014978292736	0.997071151699424
NR2F2	0.0414183198245207	1.02523695136142	-0.220085581812653	0.82341130205275
NR2F6	0.0397821192758877	0.998634769541621	-0.211391261250321	0.985340035418461
SMAD4	0.0395868703547178	0.989655350970641	-0.21035376208095	1.03298217589819
MBD1	0.0381303728514569	0.969568395081178	-0.202614334171496	1.1321170755091
SPIB	0.0379798814702096	0.988474785709098	-0.201814664282876	1.0410496742083
GATA1	0.0353183148964339	0.993051539361228	-0.187671830135953	1.02069438378406
SOX9	0.0337425207234622	1.00430151018021	-0.179298492471732	0.96184417930965
STAT6	0.0333084773802018	1.00517559321108	-0.176992105294797	0.957455989385945
NR1H3	0.0324924247604112	0.992141922935083	-0.17265582568768	1.02858255267395
PPARA	0.032301909312237	1.01015312094834	-0.171643478894437	0.930175924462582
TP63	0.0322890515122101	0.990991766445956	-0.171575156074685	1.03472026209845
FOXO1	0.0319090568228168	0.997641274348926	-0.169555968607519	1.00032099950078
ZBED1	0.0301769127688746	0.996252459051518	-0.160351830595392	1.00951402348342
GLI2	0.0284483159675553	0.996224275811926	-0.15116654170995	1.01136189830608
SMAD1	0.0274140035910682	0.999528798777671	-0.14567048967019	0.994728495269759
CDX2	0.0271429753716592	0.989323703527761	-0.14423032011215	1.04819000449127
RXRA	0.0269573898187518	0.997093465808423	-0.143244169429052	1.00815241656801
ELK1	0.0267786791176357	1.00811787166031	-0.142294549821165	0.947779451473016
ZBTB7A	0.0267177136387333	0.984909430641345	-0.141970596001921	1.07058798095786
GATA2	0.025719734368512	0.996077549052826	-0.136667608115034	1.01460655768061
TBX21	0.0253929227087017	0.963327363805537	-0.13493102066781	1.1720578432878
MAF	0.0250863789272276	0.987248728736964	-0.133302131162323	1.06033495552614
RFX5	0.0235458578584918	0.98154734467886	-0.125116225091202	1.08948329270173
RARA	0.0222476968287518	0.98920479394609	-0.118218153737094	1.05268912667454
SNAI2	0.0212679976295777	1.00238003126114	-0.113012301129715	0.984402409302533
KLF13	0.0212499323994901	1.01817069897679	-0.112916307456115	0.893405015303679
LEF1	0.0212066239028424	0.986283091981651	-0.112686177993542	1.06799034394161
ZNF263	0.0189438390933373	0.991848718280034	-0.10066236067244	1.04149396227145
ZNF592	0.0187737538311475	0.992322262674625	-0.0997585742792291	1.03917956152025
REL	0.018457882727505	1.02359302330045	-0.0980801219442	0.86175819159934
SIX5	0.018374930642565	0.982039834430937	-0.0976393373359715	1.09047623062544
KMT2A	0.0179618995747065	0.991760158280128	-0.0954446036224556	1.04253463792391
PAX5	0.016435736010102	0.991228198332355	-0.0873349893870123	1.04609152467145
HHEX	0.0161677539014317	1.01381822079746	-0.0859110060252532	0.923032435299164
RUNX3	0.0156995888763404	0.99931186995706	-0.0834233055978085	1.00444973487906
EOMES	0.0153268051792707	0.967862518846458	-0.0814424353643601	1.15788354947289
STAT3	0.0143254631182546	1.01322562876233	-0.0761215785303366	0.927537847654786
SPI1	0.0142388156149921	1.00614076157126	-0.0756611574835868	0.968010606491172
STAT5A	0.0141681839322708	1.0164830760625	-0.0752858401106906	0.908329960659701
NR5A2	0.0126551744797304	0.973873900478628	-0.0672461232158198	1.13168198788434
ARID3A	0.0117787965949046	0.980645004451643	-0.0625892917101752	1.10024598562732
NFYA	0.0114985410202611	1.00046183130977	-0.0611000905194323	1.00023627541443
HNF1B	0.0110637750806787	0.985141752991253	-0.0587898636640064	1.07877041337494
SOX10	0.010629358271605	0.975760612788234	-0.0564814919922541	1.12365440062422
POU2F2	0.00959929097552617	0.998002118841242	-0.0510079971444641	1.01398253859351
ELK4	0.00958162675518359	0.985990688500722	-0.0509141343265634	1.07509027406138
NR2F1	0.00935800321730544	1.00208169796536	-0.0497258602331373	0.992275027801646
KLF1	0.0093424983964062	1.00389762744408	-0.0496434718711038	0.982398888213526
ZNF274	0.00932953199033635	0.968029239866785	-0.0495745719486442	1.15929961588025
SOX2	0.00876195503252895	0.951225015567648	-0.0465586238003012	1.23169680248082
KLF4	0.00847232963922704	1.00611685693003	-0.0450196339653038	0.970432605070998
FOXA1	0.00764209153552642	0.993683264829889	-0.0406079765907397	1.03700477144807
REST	0.0068690222291362	0.894872029425693	-0.0365000985116774	1.44059918739998
FOS	0.00578889253726267	1.00302163817063	-0.030760585835259	0.988102347212084
MEF2C	0.00515853301710532	1.01208596950174	-0.0274110283850184	0.937412146247457
BCL11A	0.00477508327489586	0.997834318447565	-0.0253734817156255	1.01602361543763
SRF	0.00473924130513269	0.998717499851341	-0.0251830273272741	1.01137057737605
ETS1	0.00396693084975465	1.01281215849986	-0.0210791815741893	0.933398917904944
ELF3	0.00293541622844202	1.00730503657806	-0.0155979960374062	0.964917244487327
MYCN	0.00221767794300007	0.997774921482333	-0.0117841318147641	1.01663406270877
GATA4	0.000925825036387819	0.994379639018061	-0.00491958009531348	1.03436596554835
ZNF24	0.000478847038685864	0.999374386491782	-0.00254446171537054	1.00826248653905

Supplementary Table S7: Transcription activity score of the CXCL-iFibro and ECM-secreting myoFibro calculated by the Dorothea method.

Module 3 genes	Module 4 genes	Module 3 (continued)	Module 4 (continued)
CXCL1	SFRP4	AL034417	AEBP1
CXCL2	APOD	SOST	ANXA1
CXCL8	PLA2G2A	GCHFR	LAMC3
CCL13	CFD	JUN	WISP2
SERPINE1	IGF2	NCAM1	NPR1
IER3	NOV	SERPINB9	AHR
SOD2	SFRP2	HSP90AA1	TSHZ2
HSPA1A	DPT	ADH4	COL4A4
PTGS2	RPL10P9	AVPR1A	A2M
CXCL3	VCAN	RAI14	AKR1C1
ICAM1	IGFBP2	CHI3L2	CDKN2A
CCL2	FP671120	COL16A1	BMP5
G0S2	IGFBP6	COL28A1	PRSS23
IFIT2	SFRP1	EDN1	JAK2
DNASE1L3	H19	ALDH1A3	FBLN5
PDK4	CXCL14	SUPT5H	GPNMB
IL6	CCDC80	KDM6B	NPY1R
IRF1	ITGBL1	RGS2	CFH
IFIT3	RARRES1	IER5L	DEPTOR
DEPP1	CFHR1	BHLHE40	PDGFRA
RGS16	C3	CDH19	COLEC11
MT1A	SEMA3C	POLG2	PALLD
MT1X	FMO2	CEBPD	CHRDL1
CCL26	SAA1	HGF	HMCN1
GADD45B	AGTR2	LINC02551	TMEM176B
ADAMTS4	PI16	ARL4D	PRRX1
ADAMTS1	GSN	JUND	TIPARP
C11orf96	CP	NBPF9	ITGB5
CH25H	FBLN2	PHYH	CLSTN2
DDIT4	CLEC3B	DAB2	LOX
COL5A2	AKAP12	LAMB1	FILIP1L
IFIT1	S100A10	WNT2B	FREM1
XIST	OGN	SOX18	GFRA1
ICAM4	FST	TUBA1A	NEGR1
MT1M	C2orf40	NCOA7	C16orf89
FLT1	ADM	SLIT2	TMEM119
NFKBIA	FBLN1	SOCS1	SH3BP5
ENC1	EMP1	CYTOR	AOX1
HAS1	FBN1	AL590434	CGNL1
CDKN1A	F8	CITED2	CXCL12
ISG15	ARL4C	CDKN1C	ANGPTL1
SEMA4A	ITM2A	FAM43A	FMO1
BIRC3	C7	MIR4435-2H	CDC42EP3
NEAT1	PLXDC2	SAT1	PTN
CXCL6	HHIP	UBE2S	SGCD
TNFSF10	CHL1	TRHDE	CLIC5
KCNQ1OT1	FGF7	GJC1	EMILIN1
AQP1	LAMA2	CD93	CD248
BTG2	CADM3	MEST	ITGA5
VCAM1	AC037198	NEXN	HSD11B1
HBB	GPC3	LRRC2	MYH10
IFI27	ADRA2A	ASS1	MMP2
KLF2	HPD	AL034379	TMEM176A
MYC	MGST1	SESN3	MATN2
HAS2	ASPN	HMSD	SVEP1
NNMT	COL14A1	CPED1	ADAMTSL2
PDLIM3	UAP1	IER5	DAAM1
NR4A2	EXOSC7	DDX58	ABCA8
INTS6	APCDD1	RHOB	PRELP
NFKBIZ	CCDC71L	CD14	MLPH
MT2A	IGSF10	WWTR1	AHNAK
HSPA1B	TGM2	AL590648	BASP1
TIFA	THBS1	CBLN4	PODN
RNASE1	CTSK	NKX3-1	ALDH1A1
PAPPA	S100A6	FAM114A1	OLFML3
AC104581	PLCXD3	SLFN5	DHRS3
PTPRB	SRPX	ZFP36L2	FLNC
CCL14	UCHL1	GBP1	LFNG
PCDH9	EFEMP1	PDE4D	PTPRD
ADAM12	GAS1	PGM5	DIAPH3
ATF3	OMD	PAK3	SAMHD1
SOCS3	DCN	IL1R1	TNXB
FOSB	PCDH7	GABBR1	DACT1
ARHGAP18	PTGER1	PLOD2	KCNK3
KLF4	MMP19	FOS	ARL5B
LMCD1	HP	SNAI1	ANXA2
CPXM2	PDGFRL	C1QTNF2	PLTP
TNFAIP2	SVIL	ZNF154	MXRA7
DNAJB1	CCNO	IER2	CLEC4F
MT1E	PTGIS	RPL7P19	CEP131
PLEKHG2	LUM	DIRC3	SYTL5
MAFF	LTBP1	CRISPLD1	PPP1R14B
CEMIP2	PLIN5	AC092155	ADAMTS7P4
ZC3HAV1	VIM	SEMA3E	PGM5-AS1
PIM1	MEDAG	SLF2	LIMS1
HSPH1	EPHA3	FAM153A	TCF21
SGK1	CLMP	HSPA8	PDPN
LMO3	SERPINF1	AC002075	FAM89A
MAFB	DKK2	RSAD2	PREX2
NES	MARCKS	RCAN1	OSMR
NAMPT	UACA	ADGRE2	FLRT2
PHLDA1	DPEP1	CCL15-CCL14	STEAP1
ERRFI1	SEMA3D	HEXIM1	DKK3
CRYAB	PRSS35	TSC22D3	PLXNA2
NFIL3	CALM1	HIST1H4C	GXYLT2
EFNB2	GPX3	F11R	TNNI1
RPL21P12	EGR3	PTPRE	BMP6
IFI44L	TNFRSF12A	SYNPO	SYNE1
SCN7A	OSR2	LAMA4	TPT1

Supplementary Table S8: List of genes identified in module 3 and 4 by Monocle 3, represented in **Fig. S4**.

Clusters	Category	Term	Description	LogP	Log(q-value)	InTerm_InList	Symbols
Module 3	Reactome Gene Sets	R-HSA-1280215	Cytokine Signaling in Immune system	-17.2069203	-12.848	36/707	BIRC3,CDKN1A,CEBPD,FOS,GBP1,CXCL1,CXCL2,HGF,HSPA8,HSP90AA1,ICAM1,IFI27,IFIT2,IFIT1,IFIT3,IL1R1,IL6,CXCL8,IRF1,JUN,LTB,MT2A,MYC,NCAM1,NFKBIA,PIM1,PTGS2,CCL2,SOD2,VCAM1,SOCS1,SOCS3,ISG15,DDX58,XAF1,RSAD2,EDN1,LAMA4,LAMB1,GADD45B,NKX3-1,RNASE1,TGFB2,TXNRD1,WNT2B
	GO Biological Processes	GO:0001944	vasculature development	-15.7576906	-11.876	36/786	AQP1,RHOB,EDN1,EFNB2,FLT1,HAS2,HGF,IL6,CXCL8,JUN,NKX3-1,SERPINE1,PTGS2,PTPRB,SAT1,CCL2,SLC12A2,SOD2,TEK,TGFB2,TNFAIP2,ADAM12,SOCS3,KLF4,SLIT2,ADAMTS1,SEMA3E,GJC1,KLF2,CEMIP2,CLIC4,SRPX2,ERRFI1,SOX18,SEMA4A,UNC5B,DAB2,ICAM1,IL1R1,LAMB1,PAK3,SNAI1,CCL26,HAS1,CDKN1A,HTRA1
	GO Biological Processes	GO:0009636	response to toxic substance	-15.2088249	-11.452	29/506	ADH4,AQP1,RHOB,ASS1,CD14,CDKN1A,CRYAB,EDN1,FOS,HBB,HGF,ICAM1,IL6,JUN,MT1A,MT1E,MT1M,MT1X,MT2A,NR4A2,PIM1,PTGS2,RGS2,SOD2,TXNRD1,VCAM1,KLF4,KLF2,KDM6B,AVPR1A,FOSB,JUND,SLC12A2,NLGN1,CLIC4,XAF1,MYC,PDE4D,PDK4,SCN7A,CCL13,CCL14
	WikiPathways	WP4754	IL-18 signaling pathway	-14.6299146	-11.116	22/273	BIRC3,ARL4D,ATF3,FOS,CXCL2,CXCL3,ICAM1,IL6,CXCL8,IRF1,JUN,LTB,NFKBIA,PTGS2,RGS16,CCL2,TNFAIP2,BTG2,IER3,SOCS3,KLF2,NFKBIZ,AQP1,RHOB,EFNB2,CXCL1,IL1R1,MYC,SLIT2,FOSB,HSP90AA1,JUND,CXCL6,EDN1,VCAM1,FLT1,SERPINE1,CCL13,CCL14,SLC12A2,TGFB2,CH25H,CCL26,ADGRE2,MTUS1,HGF,NCAM1,SEMA3E,SEMA4A,NEXN,UNC5B,A2M,PAPPA,SERPINB9,PLOD2,HTRA1,WNT2B,ADAM12,TNFSF10,ADAMTS4,ADAMTS1,C1QTNF2,CBLN4,HMSD,TEK,F11R,CD14,HSPA1A,HSPA1B,HSPA8,ASS1,SOD2,ENC1,CCL15-CCL14,CDKN1A,RCAN1,RNASE1,GBP1,NAMPT,SCN7A,ISG15,AVPR1A,GABBR1,PDE4D,RGS3
Module 4	Canonical Pathways	M5884	NABA CORE MATRISOME	-29.3178551	-24.959	34/275	AEBP1,COL4A4,VCAN,DCN,DPT,FBLN1,FBLN2,FBN1,EFEMP1,IGFBP2,IGFBP6,CCN1,LAMA2,LTBP1,LUM,MATN2,CCN3,OMD,OGN,PCOLCE,PRELP,THBS1,TNXB,COL14A1,SRPX,CCN5,LAMC3,FBLN5,EMILIN1,ASPN,SVEP1,HMCN1,PODN,IGSF10,A2M,CTSK,FGF2,GSN,ITGA5,ITGB5,LOX,MMP2,MMP19,DDR2,PDGFRA,PLA2G2A,PLTP,SFRP2,SH3PXD2A,ADAMTSL2,PDPN,FLRT2,CCDC80
	Canonical Pathways	M5885	NABA MATRISOME ASSOCIATED	-20.5366718	-16.956	40/751	A2M,ANXA1,ANXA2,BMP5,BMP6,CTSK,FGF2,FGF7,FGF12,GPC3,IGF2,LOX,MMP2,MMP19,SERPINF1,PLXNA2,PTN,S100A6,S100A10,CXCL12,SFRP1,SFRP2,SFRP4,TGM2,CLEC3B,ANGPTL1,CXCL14,ADAMTSL2,FST,SEMA3C,FSTL1,HHIP,COLEC11,PLXDC2,CHRDL1,CCBE1,FREM1,CLEC4F,SEMA3D,INS-IGF2,EGR3,LIMS1,CCN3,THBS1,TWIST1,OSR2
	GO Biological Processes	GO:0070848	response to growth factor	-19.0334529	-15.520	38/736	AGTR2,ANXA1,BMP5,BMP6,DCN,EGR3,FBN1,FGF2,FGF7,FGF12,GAS1,GPC3,CCN1,ITGA5,ITGB5,LIMS1,LOX,LTBP1,LUM,PDGFRA,PTN,SFRP1,SFRP2,SFRP4,THBS1,CLEC3B,TWIST1,ADAMTSL2,FST,EMILIN1,FSTL1,FLRT2,DKK3,ASPN,HHIP,RASL11B,CHRDL1,CCBE1,JAK2,CCN3,DACT1,VIM,C3,CP,VCAN,IGF2,IGFBP2,IGFBP6,MMP2,PRSS23,ZBTB16,HIF3A
	GO Biological Processes	GO:0001944	vasculature development	-16.3670837	-13.008	36/786	ADM,AHR,ANXA1,ANXA2,APOD,C3,DCN,EGR3,FGF2,GPC3,CCN1,ITGA5,LOX,MMP2,MMP19,CCN3,NPR1,PDGFRA,SERPINF1,PRRX1,PTGIS,PTN,SFRP1,SFRP2,TCF21,THBS1,TWIST1,ANGPTL1,GPNMB,SEMA3C,PDPN,EMILIN1,TIPARP,TNFRSF12A,HIF3A,CCBE1,ADRA2A,FGF7,JAK2,DDR2,CXCL12,CXCL14,AKAP12,SEMA3D

Supplementary Table S9: Functional enrichment results of module 3 and 4 identified by Monocle 3, represented in **Fig. S5** and characterized by the Metascape tool. The p-value is the result of a hypergeometric enrichment test. Q-value correspond to the Bonferonni corrected p-value (or False Discovery Rate).

CXCL-ifibro signature
TMEM176A
FMO3
DPEP1
HGF
BIRC3
TYMP
SLC2A3
VMP1
CNN2
GGT5
MYL12A
CRISPLD2
HMOX2
SFRP1
TMEM176B
CXCL12
CCL2
C7
RND3
ANGPTL1
NID1
COLEC11
CTGF
C2orf40
SRGN
G0S2
SDC4
FOSB
ZFP36
RFTN1
EDNRB
CCL21
THBS1
DUSP6
FBLN5
ABCA8
CYR61
MYO10
GFRA1
GRAMD2B
IL34
PCSK7
PTGER1
DNAJB4
VCAM1
CTSS
NPY1R
ALDH7A1
CYB5A
SLC43A2
FILIP1L
FOS
SERPINB9
NEGR1
CCL19
JUN
ODF3B
MYADM
CCBE1
EMID1
TSPAN4
CEBPD
STON1
CFHR1
SELENOP

Supplementary Table S10: list of 65 genes characterizing the CXCL-iFibro

threshold of CXCL-iFibro expression	patients under the treshold (n=)	patients above the treshold (n=)	pvalue
3.994576851	1	133	0.672318993
4.049233603	2	132	0.55937031
4.103852429	3	131	0.425875596
4.138080132	4	130	0.334015
4.144337462	5	129	0.277196069
4.154852764	6	128	0.242733685
4.179118283	7	127	0.191900119
4.192646943	8	126	0.154944334
4.193021692	9	125	0.419242809
4.218555543	10	124	0.334722681
4.233613247	11	123	0.267541263
4.240401717	12	122	0.228079967
4.247901365	13	121	0.185467344
4.276880152	14	120	0.148087533
4.288439021	15	119	0.118023422
4.307300259	16	118	0.096763059
4.327062328	17	117	0.07677645
4.330907298	18	116	0.062667692
4.345900219	19	115	0.049430693
4.346963994	20	114	0.040122241
4.353603269	21	113	0.032464055
4.361116885	22	112	0.026994852
4.380472356	23	111	0.077781532
4.384825407	24	110	0.061662989
4.388353667	25	109	0.051197616
4.388588052	26	108	0.041529818
4.390482221	27	107	0.033121065
4.406308223	28	106	0.026637675
4.407848118	29	105	0.020639824
4.410518225	30	104	0.015893446
4.415291826	31	103	0.012158771
4.428951552	32	102	0.00958344
4.436886325	33	101	0.008330444
4.440349742	34	100	0.006273314
4.444604941	35	99	0.004799401
4.448490678	36	98	0.00364728
4.454297575	37	97	0.011777028
4.456651545	38	96	0.009855208
4.461746859	39	95	0.00740094
4.46272144	40	94	0.005510429
4.513309585	41	93	0.004167339
4.526161386	42	92	0.010100558
4.532306882	43	91	0.007534306
4.542323781	44	90	0.005878005
4.543100319	45	89	0.005585846
4.544352128	46	88	0.015020463
4.550813809	47	87	0.01128135
4.555103002	48	86	0.008386682
4.562772199	49	85	0.006168113
4.564443431	50	84	0.005518937
4.566519845	51	83	0.004107252
4.567650654	52	82	0.002944627
4.583933682	53	81	0.006802804
4.598525377	54	80	0.005220916
4.609860398	55	79	0.00447098
4.614502068	56	78	0.003272132
4.625763634	57	77	0.002300782
4.636642576	58	76	0.005978145
4.650333639	59	75	0.004259872
4.654816435	60	74	0.010663198
4.65764841	61	73	0.007711619
4.683136373	62	72	0.006380301
4.694719458	63	71	0.01712229
4.701500668	64	70	0.012538088
4.70296636	65	69	0.01043948
4.703017529	66	68	0.007464541

threshold of CXCL-iFibro expression (continued)	patients under the treshold (n=) (continued)	patients above the treshold (n=) (continued)	pvalue (continued)
4.7039561975	67	67	0.0069821298
4.7084402772	68	66	0.0051450023
4.7125983791	69	65	0.0035534573
4.7203643172	70	64	0.0024081533
4.7332848069	71	63	0.0069663555
4.7400424316	72	62	0.004816166
4.7404987047	73	61	0.0114780534
4.7431529147	74	60	0.0080423482
4.7631082806	75	59	0.0057046721
4.7697090387	76	58	0.004273306
4.7701861705	77	57	0.0109270252
4.7827743807	78	56	0.007525392
4.7846094479	79	55	0.0050627932
4.7908918052	80	54	0.0033208264
4.7959396917	81	53	0.0022063725
4.8119087443	82	52	0.0014305903
4.8122434808	83	51	0.0009034207
4.8128788534	84	50	0.0007960659
4.8172760435	85	49	0.0024776657
4.8275569794	86	48	0.0075940421
4.8342143864	87	47	0.0051017422
4.8428930655	88	46	0.0128071831
4.8600968706	89	45	0.0094396552
4.8610898446	90	44	0.0251106349
4.8710174312	91	43	0.0180404368
4.8837535964	92	42	0.0119386615
4.8891128527	93	41	0.0076261908
4.8906619628	94	40	0.0068697605
4.8996384614	95	39	0.014153139
4.916601815	96	38	0.0089164538
4.9168513199	97	37	0.005372689
4.9193672137	98	36	0.0178248478
4.9520984774	99	35	0.0460202448
4.9603330058	100	34	0.034355197
4.9619385644	101	33	0.0778581773
4.9671513482	102	32	0.0554748034
4.9982941764	103	31	0.0367751085
5.0015391462	104	30	0.0948141151
5.0100969342	105	29	0.0672417758
5.0150874637	106	28	0.1656957773
5.0318670016	107	27	0.1247026012
5.0450718521	108	26	0.0907652022
5.0627498172	109	25	0.2169169166
5.0813711613	110	24	0.1670281213
5.1430087675	111	23	0.1560587344
5.1558510982	112	22	0.1285684322
5.1652292828	113	21	0.2528522393
5.1652988774	114	20	0.1816408889
5.1744843262	115	19	0.1369772475
5.1810905634	116	18	0.1011410147
5.1964788644	117	17	0.2349857271
5.1993264349	118	16	0.4867674722
5.2427194131	119	15	0.3684299373
5.2459201053	120	14	0.6467901422
5.2543920145	121	13	0.5219561362
5.3086592465	122	12	0.8664033713
5.3096852439	123	11	0.8664033713
5.3131353012	124	10	0.7302957551
5.3166573398	125	9	0.8758148396
5.3544296605	126	8	0.974279623
5.3605535174	127	7	0.8769431088
5.3981439598	128	6	0.6969283303
5.4083916213	129	5	0.7881508509
5.5049793879	130	4	0.9877814207
5.565625243	131	3	0.749608269
5.652771791	132	2	0.3696302706

Supplementary Table S11: Results of the iterative method determining the difference by Log-rank test between 2 groups of patients, according to their expression below or above a threshold of CXCL-iFibro expression score. The threshold with the lowest p-value is highlighted in yellow. The p value is calculated with log rank test.

threshold of FOLR2 expression	patients under the treshold (n=)	patients above the treshold (n=)	pvalue	threshold of FOLR2 expression (continued)	patients under the treshold (n=) (continued)	patients above the treshold (n=) (continued)	pvalue (continued)
1.537286095	1	133	0.576216382	3.613706462	67	67	0.017149395
1.652543219	2	132	0.481975752	3.619296732	68	66	0.012771724
1.685853878	3	131	0.3660513	3.639472353	69	65	0.03189529
1.822274605	4	130	0.29002742	3.649549318	70	64	0.025622358
1.971223086	5	129	0.23509046	3.681007781	71	63	0.052349631
2.031459298	6	128	0.185629556	3.699690002	72	62	0.113906861
2.068504087	7	127	0.149909821	3.723560736	73	61	0.206344181
2.071305288	8	126	0.119010867	3.734858964	74	60	0.170549764
2.078784686	9	125	0.09632066	3.741227814	75	59	0.16343637
2.136041604	10	124	0.081922612	3.74297325	76	58	0.144144108
2.195569458	11	123	0.067091438	3.746156192	77	57	0.114360484
2.318501408	12	122	0.053184846	3.74796761	78	56	0.089329961
2.330416486	13	121	0.163296721	3.783236241	79	55	0.0701622
2.337006233	14	120	0.130144909	3.796609677	80	54	0.053036523
2.413205202	15	119	0.105446476	3.81292147	81	53	0.0426054
2.415970691	16	118	0.086347274	3.81632088	82	52	0.04043056
2.419528299	17	117	0.06835735	3.836939966	83	51	0.032041552
2.463525797	18	116	0.053926595	3.861065769	84	50	0.070469147
2.491361262	19	115	0.043822495	3.88138085	85	49	0.052729996
2.499699854	20	114	0.034328563	3.899791123	86	48	0.039655373
2.507852018	21	113	0.030348075	3.902085841	87	47	0.070452969
2.539888245	22	112	0.023622267	3.902901554	88	46	0.052023501
2.546607427	23	111	0.019392824	3.91020885	89	45	0.111614939
2.558191079	24	110	0.014966268	3.97154551	90	44	0.206463565
2.61952601	25	109	0.011926125	4.069640108	91	43	0.162755707
2.62434585	26	108	0.009110857	4.082975552	92	42	0.282107846
2.64816778	27	107	0.007194681	4.107152727	93	41	0.22654009
2.693822409	28	106	0.005434574	4.125451093	94	40	0.181788617
2.700145602	29	105	0.004181438	4.137826802	95	39	0.15152322
2.724589733	30	104	0.003117589	4.154162529	96	38	0.114536516
2.757293062	31	103	0.002506846	4.15585704	97	37	0.088000077
2.791843957	32	102	0.001956451	4.171346528	98	36	0.081166185
2.792455626	33	101	0.001431897	4.186304417	99	35	0.061477153
2.805746366	34	100	0.001069089	4.187633691	100	34	0.139676162
2.83068144	35	99	0.003367994	4.192556397	101	33	0.121207998
2.890593241	36	98	0.002470121	4.193462423	102	32	0.092053841
2.939614785	37	97	0.00644117	4.327361677	103	31	0.066757001
2.985945423	38	96	0.00608906	4.333635727	104	30	0.04786807
3.008902509	39	95	0.005481823	4.352258526	105	29	0.038280368
3.031718403	40	94	0.004038797	4.361792977	106	28	0.024336143
3.03800912	41	93	0.002947217	4.364812245	107	27	0.01528596
3.0586604	42	92	0.002129068	4.40777685	108	26	0.048599045
3.066904523	43	91	0.001521788	4.455152472	109	25	0.030614915
3.068115415	44	90	0.001110553	4.479376663	110	24	0.019531432
3.071631661	45	89	0.000777066	4.504869954	111	23	0.011795562
3.078400797	46	88	0.000555821	4.633803244	112	22	0.006066574
3.096895401	47	87	0.000393437	4.651457495	113	21	0.003259674
3.097547894	48	86	0.000309846	4.652951781	114	20	0.012120696
3.104705622	49	85	0.00025257	4.707099225	115	19	0.006173396
3.129213677	50	84	0.000168225	4.716232233	116	18	0.023439634
3.135215731	51	83	0.000630714	4.820603529	117	17	0.069015048
3.180107728	52	82	0.001627922	4.837609349	118	16	0.15476102
3.183420077	53	81	0.001328932	4.866307716	119	15	0.321150472
3.263549725	54	80	0.000942324	4.884881977	120	14	0.574564683
3.31767196	55	79	0.002454103	4.925186124	121	13	0.978655402
3.349553128	56	78	0.001794394	4.967523874	122	12	0.944033509
3.369342209	57	77	0.00122899	4.972074634	123	11	0.812228753
3.375996532	58	76	0.003785861	5.038131601	124	10	0.666431867
3.400482025	59	75	0.010907763	5.051540014	125	9	0.494656103
3.404359287	60	74	0.025033091	5.060276561	126	8	0.906688489
3.443331591	61	73	0.058181177	5.258923935	127	7	0.755580635
3.451848483	62	72	0.044856943	5.301403802	128	6	0.552741405
3.512454878	63	71	0.035470846	5.408863051	129	5	0.940922718
3.555580712	64	70	0.028790206	5.410108676	130	4	0.940922718
3.555745816	65	69	0.027006774	5.638151627	131	3	0.327548173
3.598116853	66	68	0.020028671	5.980847622	132	2	0.426683627

Supplementary Table S12: Results of the iterative method determining the difference by Log-rank test between 2 groups of patients, according to their expression below or above a threshold of FOLR2 expression score. The threshold with the lowest p-value is highlighted in yellow. The p value is calculated with log rank test.

Genes
ABCB1
CCND1
NRXN1
CD44
HDAC7
NOL4L
SPTBN1
ADD3
AREG
ASAP2
ATP8B1
CD9
CDK6
CEP112
CHN2
DAAM1
EHBP1
EPB41L1
EPB41L2
ERI3
FGD5
FOXP1
FRMD3
GLS
GOLIM4

Supplementary Table S13
List of TCF4-target genes used in Fig. S6.

Immunohistochemistry (IHC)		
**Antibodies**	**Reference**	**Dilution**
Rabbit Recombinant Monoclonal FAP antibody	Abcam #ab207178	1/100
Rabbit Recombinant Monoclonal SFRP1 antibody	Abcam #ab126613	1/200
Rabbit Recombinant Monoclonal SFRP4 antibody	Abcam #ab154167	1/200
Anti-RAMP1 Antibody	EMD Millipore #MABS1904	1/400

Immunofluorescence (IF)		
**Antibodies for *in vitro* experiments**	**Reference**	**Dilution**
Mouse monoclonal anti-aSMA antibody	DAKO clone 1A4 #M0851	1/200
Rabbit monoclonal anti-SFRP1 antibody	Abcam #ab126613	1/50
Rabbit Monoclonal anti-SFRP4 antibody	Abcam #ab154167	1/400
Rabbit monoclonal anti-E-Cadherin	Cell Signaling Technology #4065	1/200
Mouse monoclonal anti-β-catenin	Bio SB #BSD 5088	1/1000
Secondary antibody goat anti rabbit alexa fluor 555	Invitrogen #A21428	1/400
Secondary antibody goat anti mouse alexa fluor 488	Invitrogen A11001	1/400
**Antibodies for human tissue sections analysis**	**Reference**	**Dilution**
Recombinant Anti-CD68 antibody	Abcam #ab955	1/200
Anto-α-SMA antibody	Dako clone 1A4 #M0851	1/400
FOLR2 monoclonal antibody	Invitrogen #MA5-26933	1/200
Human TREM2 antibody	R&D # MAB17291	1/100
Collagen I monoclonal antibody	Invitrogen #MA1-26771	1/400
Alexa fluor 488 goat anti mouse	Invitrogen #A11001	1/400
Alexa fluor-555 goat anti rabbit	Invitrogen #A21428	1/400
Alexa fluor-647 goat anti rabbit	Invitrogen #A21245	1/400
Alexa fluor-555 goat anti rat	Invitrogen #A21434	1/400

Flow Cytometry (FACS)		
**Antibody pool for CD14+ monocytes characterization after co-culture**	**Reference**	**Dilution**
BV510 Anti-human CD14	BD Biosciences #563079	1/50
BV650 Anti-human CD16	BD Biosciences #563692	1/50
BV510 Anti-human CD206	Biolegend #321136	1/50
PE Anti-human FOLR2	Biolegend #391704	1/50
Biotinyl-Anti Human TREM2	R&D systems #BAF1828	1/50
PECY5 streptavidin	Biolegend #405205	1/100
**IgG controls**	**Reference**	**Dilution**
BV510 IgG1κ mouse isotype control	BD Biosciences #562946	1/50
BV650 IgG1κ mouse isotype control	BD Biosciences #563231	1/50
BV711 IgG1κ mouse isotype control	BD Biosciences #563044	1/50
PE IgG1κ mouse isotype control	Biolegend #400112	1/50
Goat IgG isotype control	R&D systems #AB-108-C	1/50

Western Blot		
**Antibodies**	**Reference**	**Dilution**
Rabbit Recombinant Monoclonal FAP antibody	Abcam #ab207178	1/1000
Rabbit Recombinant Monoclonal SFRP1 antibody	Abcam #ab126613	1/500
Rabbit Recombinant Monoclonal SFRP4 antibody	Abcam #ab154167	1/1000
Anto-α-SMA antibody	Dako clone 1A4 #M0851	1/1000
Actin Monoclonal antibody	Sigma #A5441	1/10000
eIF4A1 antibody	Cell signaling #2490	1/1000
β-catenin antibody	Cell signaling #9562	1/1000
Anti-histone H3 antibody	Abcam #ab1791	
secondary antibody anti-mouse	Jackson ImmunoResearch Laboratories, INC., #115-035-003	1/10000
secondary antibody anti-rabbit	Jackson ImmunoResearch Laboratories, INC., #115-035-003	1/10000

Table S14: List of Antibodies used in our study and their dilutions
