# Supplementary material for: WNT-dependent interaction between inflammatory fibroblasts and FOLR2+ macrophages promotes fibrosis in chronic kidney disease
Source: Nat Commun. 2024 Jan 25;15:743. doi: 10.1038/s41467-024-44886-z (PMC10810789; doi:10.1038/s41467-024-44886-z)
Supplement: Supplementary file 3 — Reporting Summary [file 41467_2024_44886_MOESM3_ESM.pdf]

nature portfolio

Corresponding author(s): Fatima Mechta-Grigoriou

Last updated by author(s): Dec 22, 2023

Reporting Summary

Nature Portfolio wishes to improve the reproducibility of the work that we publish. This form provides structure for consistency and transparency in reporting. For further information on Nature Portfolio policies, see our Editorial Policies and the Editorial Policy Checklist.

Statistics

For all statistical analyses, confirm that the following items are present in the figure legend, table legend, main text, or Methods section.

n/a	Confirmed	
☐	☒	The exact sample size (*n*) for each experimental group/condition, given as a discrete number and unit of measurement
☐	☒	A statement on whether measurements were taken from distinct samples or whether the same sample was measured repeatedly
☐	☒	The statistical test(s) used AND whether they are one- or two-sided<br>*Only common tests should be described solely by name; describe more complex techniques in the Methods section.*
☐	☒	A description of all covariates tested
☐	☒	A description of any assumptions or corrections, such as tests of normality and adjustment for multiple comparisons
☐	☒	A full description of the statistical parameters including central tendency (e.g. means) or other basic estimates (e.g. regression coefficient) AND variation (e.g. standard deviation) or associated estimates of uncertainty (e.g. confidence intervals)
☐	☒	For null hypothesis testing, the test statistic (e.g. *F*, *t*, *r*) with confidence intervals, effect sizes, degrees of freedom and *P* value noted<br>*Give P values as exact values whenever suitable.*
☐	☒	For Bayesian analysis, information on the choice of priors and Markov chain Monte Carlo settings
☒	☐	For hierarchical and complex designs, identification of the appropriate level for tests and full reporting of outcomes
☐	☒	Estimates of effect sizes (e.g. Cohen's *d*, Pearson's *r*), indicating how they were calculated

Our web collection on statistics for biologists contains articles on many of the points above.

Software and code

Policy information about availability of computer code

Data collection	Microsoft Excel 2021
Data analysis	data analysis has been performed using R version 4.2.2 Patched (2022-11-10 r83330)<br>Platform: x86_64-pc-linux-gnu (64-bit)<br>Running under: Ubuntu 22.04.3 LTS<br><br>Packages: dorothea_1.10.0;BayesPrism_2.0; monocle3_1.3.1;rstatix_0.7.2;Scpubr_2.0.1; ggplot2_3.4.3; survival 3.5-7; tidyverse_2.0.0; Seurat_4.4.0<br><br>A code availability section has been introduced:<br><br>CODE AVAILABILITY<br>All the codes used for this study are available on Figshare under the DOI 10.6084/m9.figshare.24049350 (https://figshare.com/search?q=10.6084%2Fm9.figshare.24049350). They can be also accessible on GitHub (https://github.com/StressAndCancerLab/).

For manuscripts utilizing custom algorithms or software that are central to the research but not yet described in published literature, software must be made available to editors and reviewers. We strongly encourage code deposition in a community repository (e.g. GitHub). See the Nature Portfolio guidelines for submitting code & software for further information.

Data

Policy information about availability of data

All manuscripts must include a data availability statement. This statement should provide the following information, where applicable:

- Accession codes, unique identifiers, or web links for publicly available datasets
- A description of any restrictions on data availability
- For clinical datasets or third party data, please ensure that the statement adheres to our policy

DATA AVAILABILITY
New generated data, as well as source data and codes have been deposited in Figshare. Raw counts for bulk RNAseq for primary human fibroblasts can be downloaded on Figshare under the DOI 10.6084/m9.figshare.24049380 (https://figshare.com/search?q=10.6084%2Fm9.figshare.24049380). Spatial transcriptomics processed data for the 2 patients with kidney fibrosis can be downloaded on Figshare under the DOI 10.6084/m9.figshare.24049410 (https://figshare.com/search?q=10.6084%2Fm9.figshare.24049410). Raw data have been deposited on the European Genome Phenome Archive (EGA) and can be requested through the Data Access Committee EGAC00001000581.
Publicly available single cell RNAseq data for 12 patients with kidney disease from Kuppe et al. {Kuppe, 2021 #4} including matrix count and annotations were downloaded from Zenodo data archive (https://zenodo.org/record/4059315, DOI: 10.5281/zenodo.4059315). Publicly available bulk RNAseq data from 15 mice undergoing unilateral ureteral obstruction (UUO) were downloaded on the Gene Expression Omnibus under the number GSE118339 {Pavkovic, 2019 #98}. The publicly available single cell RNAseq dataset from mice UUO was downloaded in the National Center for Biotechnology Information Gene Expression Omnibus database (accession number GSE140023). Source data are provided with this paper under the DOI 10.6084/m9.figshare.24049305 (https://figshare.com/search?q=10.6084%2Fm9.figshare.24049305%20). Uncropped blots are available at 10.6084/m9.figshare.24049938 (https://figshare.com/search?q=10.6084%2Fm9.figshare.24049938).

Research involving human participants, their data, or biological material

Policy information about studies with human participants or human data. See also policy information about sex, gender (identity/presentation), and sexual orientation and race, ethnicity and racism.

Reporting on sex and gender	We used the biological variable "Sex". It was self reported
Reporting on race, ethnicity, or other socially relevant groupings	This is provided in Table 1 and Table S5
Population characteristics	This is provided in Table 1 and Table S5
Recruitment	Restrospective study, on historical cohort. All PKD patients who had a kidney sample stored in the pathology department were included. For kidney biopsies, patients with vascular nephropathies and kidney fibrosis were selected by a pathologist to include patients with a range of fibrosis fro 0 to 80%, blindy from all other authors.
Ethics oversight	All the performed studies were validated by the local ethics commitee from Institut Curie (Poesie DATA220128) and Assistance-Publique Hopitaux de Paris (APHP): Comité d'Ethique de la Recherche (CER) Paris Nord, Institutional Review Board -IRB 00006477- of HUPNVS, Paris 7 University, AP-HP, file CER-2022-174).

Note that full information on the approval of the study protocol must also be provided in the manuscript.

Field-specific reporting

Please select the one below that is the best fit for your research. If you are not sure, read the appropriate sections before making your selection.

☒ Life sciences ☐ Behavioural & social sciences ☐ Ecological, evolutionary & environmental sciences

For a reference copy of the document with all sections, see nature.com/documents/nr-reporting-summary-flat.pdf

Life sciences study design

All studies must disclose on these points even when the disclosure is negative.

Sample size	For human tissue samples, the sample size was dependent of the material availability from the pathology department. This is the same for spatial transcriptomics experiment.<br>In vitro, we were able to generate 3 pairs of fibroblasts culture either on collagen or plastic. These experiment were dependent of the number of patient undergoing a kidney explant for a PKD (approximately 4 per year in our institution).<br>Sample size were determined as the available material for generation of human in vitro primary cultures of fibroblast (n=3), as well as the available sample from helathy blood donors (n=6 usually)
Data exclusions	No data were excluded
Replication	All the experiments were performed independantly

Randomization	For all in vitro and coculture studies, all the fibroblast cell lines available (3) were cocultured in the same time by the same healthy PBMC donors (n=1-2 depending on the day of the experiment). Healthy donors were randomly selected and blind to the investigator. Etablissement Francais du Sang was in charge to collect and deliver blood from healthy donor to Institut Curie
Blinding	For quantification of immunohistochemistry or immunofluorescence in humans, the investigator was blind from the pathological assessment, and especially the degree of fibrosis<br>For quantification of MFI in vitro, FACS analysis the investigator was blind from treatment administrated to cells.

Reporting for specific materials, systems and methods

We require information from authors about some types of materials, experimental systems and methods used in many studies. Here, indicate whether each material, system or method listed is relevant to your study. If you are not sure if a list item applies to your research, read the appropriate section before selecting a response.

Materials & experimental systems

n/a	Involved in the study
☐	☒ Antibodies
☐	☒ Eukaryotic cell lines
☒	☐ Palaeontology and archaeology
☒	☐ Animals and other organisms
☐	☒ Clinical data
☒	☐ Dual use research of concern
☒	☐ Plants

Methods

n/a	Involved in the study
☒	☐ ChIP-seq
☐	☒ Flow cytometry
☒	☐ MRI-based neuroimaging

Antibodies

Antibodies used	CD206 Biolegend BV 711 321136, clone 15-2<br>CD14 BD BV510 563079, clone MϕP9<br>CD16 BD BV650 563692, clone 3G8<br>TREM2 R&D biotinylated BAF1828, polyclonal<br>FOLR2 Biolegend PE 391704, Clone 94b/FOLR2<br>strepta Biolegend PE/Cy5 405205<br><br>FAP Abcam Recombinant Anti-Fibroblast activation protein, alpha antibody [EPR20021] (ab207178)<br>SFRP1 Abcam Recombinant Anti-SFRP1 antibody [EPR7003] (ab126613)<br>SFRP4 Abcam Recombinant Anti-SFRP4 antibody [EPR9389] (ab154167)<br>CD68 Abcam Recombinant Anti-CD68 antibody [KP1] (ab955)<br>aSMA Dako Dako mouse clone 1A4 M0851<br>FOLR2 ThermoFisher Invitrogen mouse MA5-26933 clone OTI4G6<br>TREM2 R&D rat MAB17291<br>Beta-Catenin clone 14 Mouse Mab BioSB BSB 5088<br>Collagen I invitrogen mouse MA1-26771<br>RAMP1 EMD Millipore MABS1904 clone 4F8.1<br><br>EIF4A1 (1:1000, Cell signaling #2490),<br>Histone H3 (1:10000, abcam #ab1791)<br>B-catenin (1:1000, cell signaling #9562)
Validation	CD206 Biolegend BV 711 321136, clone 15-2 RRID AB_2687200 Oostindie SC, et al. 2022. Nat Biotechnol.<br>CD14 BD BV510 563079, clone MϕP9 RRID AB_2737993<br>CD16 BD BV650 563692, clone 3G8 RRID AB_2744298 Single-Cell Analysis of Human Mononuclear Phagocytes Reveals Subset-Defining Markers and Identifies Circulating Inflammatory Dendritic Cells. Dutertre et al. Immunity 2019<br>TREM2 R&D biotinylated BAF1828, polyclonal RRID AB_2208688<br>FOLR2 Biolegend PE 391704, Clone 94b/FOLR2 RRID AB_2721335 Sharma A, et al. 2020. Cell. 183(2):377-394.e21<br>strepta Biolegend PE/Cy5 405205 Muliaditan T, et al. 2021. Cell Rep Med. 2:100457<br><br>FAP Abcam Recombinant Anti-Fibroblast activation protein, alpha antibody [EPR20021] (ab207178) RRID AB_2864720 Aghajanian H et al. Targeting cardiac fibrosis with engineered T cells. Nature 573:430-433 (2019).<br>SFRP1 Abcam Recombinant Anti-SFRP1 antibody [EPR7003] (ab126613) RRID AB_11128257 Zhang R et al. A CRISPR screen defines a signal peptide processing pathway required by flaviviruses. Nature 535:164-8<br>SFRP4 Abcam Recombinant Anti-SFRP4 antibody [EPR9389] (ab154167) Xia L et al. CHD4 Has Oncogenic Functions in Initiating and Maintaining Epigenetic Suppression of Multiple Tumor Suppressor Genes. Cancer Cell 31:653-668.e7<br>CD68 Abcam Recombinant Anti-CD68 antibody [KP1] (ab955) RRID AB_307338 Zhang L et al. Single-Cell Analyses Inform Mechanisms of Myeloid-Targeted Therapies in Colon Cancer. Cell 181:442-459.e29 (2020)<br>aSMA Dako Dako mouse clone 1A4 M0851 RRID AB_2223500 Rizeq MN, van de Rijn M, Hendrickson MR, Rouse RV. A comparative immunohistochemical study of uterine smooth muscle neoplasms with emphasis on the epithelioid variant. Hum Pathol 1994,25:671-7

FOLR2 ThermoFisher Invitrogen mouse MA5-26933 clone OTI4G6 RRID AB_2723188
TREM2 R&D rat MAB17291 RRID AB_2208679 Generation of cryopreserved macrophages from normal and genetically engineered human pluripotent stem cells for disease modelling ,
Beta-Catenin clone 14 Mouse Mab BioSB BSB 5088 1. Alman BA, et al. Am J Pathol. 1997;Aug.151(2):329-34
Collagen I invitrogen mouse MA1-26771 RRID AB_2081889 eLife
RAMP1 EMD Millipore MABS1904 clone 4F8.1 Specificity https://www.merckmillipore.com/GB/en/product/Anti-RAMP1-Antibody-clone-4F8.1,MM_NF-MABS1904-25UG?ReferrerURL=https%3A%2F%2Fwww.google.com%2F#anchor_Product%20Information

B-catenin (1:1000, cell signaling #9562): RRID:AB_331149
Histone H3 Abcam 1791: RRID:AB_302613
EIF4A1 (1:1000, Cell signaling #2490), RRID:AB_823487

Eukaryotic cell lines

Policy information about cell lines and Sex and Gender in Research

Cell line source(s)	For primary cell lines, 2 were derived from female patients, 1 from male
Authentication	None of the cell lines were authenticated (primary cell lines)
Mycoplasma contamination	All cell lines were regularly tested negative for mycoplasma contamination
Commonly misidentified lines (See ICLAC register)	No commonly misidentified cell lines were used in the study

Clinical data

Policy information about clinical studies

All manuscripts should comply with the ICMJE guidelines for publication of clinical research and a completed CONSORT checklist must be included with all submissions.

Clinical trial registration	Retrospective study
Study protocol	For the Neptune study, the design can be found on the following reference :<br>Design of the Nephrotic Syndrome Study Network (NEPTUNE) to evaluate primary glomerular nephropathy by a multidisciplinary approach, Gadegbeku et al., DOI 10.1038/ki.2012.428.
Data collection	NEPTUNE is a multicenter observational, prospective cohort study of children and adults with proteinuric glomerular disease, for which comprehensive clinical and molecular phenotyping data was collected at 21 sites at the time of first clinically indicated renal biopsy {Gadegbeku, 2013 #71}. Biospecimens were collected after informed consent and with approval of the local ethics committee {Ju, 2015 #45}.<br>For the patients not in the NEPTUNE cohort (PKD patients, kidney biopsy cohort, spatail transcriptomics): data were pseudo-anonymized and data collection was perfromed on a securized excel spreadsheet (password). Data were stored on a securized server at the institut Curie.
Outcomes	Primary oucome was defined as a classical outcome in chronic kidney disease progression studies, such as reach of end-stage renal disease or decrease of eGFR of more than 40%

Plants

Seed stocks	*Report on the source of all seed stocks or other plant material used. If applicable, state the seed stock centre and catalogue number. If plant specimens were collected from the field, describe the collection location, date and sampling procedures.*
Novel plant genotypes	*Describe the methods by which all novel plant genotypes were produced. This includes those generated by transgenic approaches, gene editing, chemical/radiation-based mutagenesis and hybridization. For transgenic lines, describe the transformation method, the number of independent lines analyzed and the generation upon which experiments were performed. For gene-edited lines, describe the editor used, the endogenous sequence targeted for editing, the targeting guide RNA sequence (if applicable) and how the editor was applied.*
Authentication	*Describe any authentication procedures for each seed stock used or novel genotype generated. Describe any experiments used to assess the effect of a mutation and, where applicable, how potential secondary effects (e.g. second site T-DNA insertions, mosiacism, off-target gene editing) were examined.*

Flow Cytometry

Plots

Confirm that:

☒ The axis labels state the marker and fluorochrome used (e.g. CD4-FITC).

☒ The axis scales are clearly visible. Include numbers along axes only for bottom left plot of group (a 'group' is an analysis of identical markers).

☒ All plots are contour plots with outliers or pseudocolor plots.

☒ A numerical value for number of cells or percentage (with statistics) is provided.

Methodology

Sample preparation	For macrophage analysis, 3x104 fibroblasts were plated in 24 wells plate coated or not with type I collagen for 24h. Then 1.5x105 CD14+ PBMC in DMEM supplemented with 1% heat inactivated FBS and 1% Penicillin streptomycin were added. After 24 h adherent and non-adherent CD14+ monocytes were harvested, washed and stained first with LIVE/DEAD dye (1:1000, Thermo Fischer, #L34955) for 10 min at room temperature (RT) in PBS to exclude dead cells. Cells suspensions were then incubated for 20 min at RT with antibody mix containing anti-CD14-BV510 (1:50, BD biosciences 563079), anti-CD16-BV650 (1:50, BD biosciences 563692), anti-CD206-BV711 (1:50, BioLegend 321136), anti-FOLR2-PE (1:50, BioLegend 391704), anti-TREM2-biotinilated (1:50, R&D BAF1828) followed by incubation with streptavidin-PECy5 (1:100, BioLegend 405205) for 15 min. Isotype controls were BV510 mouse IgG1k (1:50, BD biosciences 56294, BV650 mouse IgG1k (1:50, BD biosciences 563231), BV711 mouse IgG1k (1 :50, BD biosciences 56344), mouse IgG1k (1:50, BioLegend 400112), Goat IgG control (1:50, R&D AB-108-C).
Instrument	LSRFortessaTM analyzer (BD biosciences)
Software	FlowJo 10.5.2
Cell population abundance	The whole populaiton from the cell culture was analyzed
Gating strategy	Gating strategy consisted in selected cells, then singlets using FSC/A-FSC-H, then live cells (live-dead dye). CD14+ CD16+ cells were gated, followed by CD206+ cells. Finally FOLR2+ cells were quantified

☒ Tick this box to confirm that a figure exemplifying the gating strategy is provided in the Supplementary Information.
